# The Regulation of Trace Metal Elements in Cancer Ferroptosis

**DOI:** 10.1002/adbi.202400821

**Published:** 2025-04-09

**Authors:** Xiaoyan Wang, Yuanyuan Xue, Lei Chang, Xuena Zhu, Wenjun Liu, Tingbo Liang

**Affiliations:** ^1^ Department of Hepatobiliary and Pancreatic Surgery The First Affiliated Hospital Zhejiang University School of Medicine Hangzhou 310003 China; ^2^ Zhejiang Provincial Key Laboratory of Pancreatic Disease The First Affiliated Hospital Zhejiang University School of Medicine Hangzhou 310003 China; ^3^ MOE Joint International Research Laboratory of Pancreatic Diseases The First Affiliated Hospital Zhejiang University School of Medicine Hangzhou 310003 China; ^4^ Department of Pathology The First Affiliated Hospital of Zhejiang University School of Medicine Hangzhou 310003 China; ^5^ Zhejiang Clinical Research Center of Hepatobiliary and Pancreatic Diseases Hangzhou 310003 China; ^6^ The Innovation Center for the Study of Pancreatic Diseases of Zhejiang Province Hangzhou 310003 China; ^7^ Zhejiang University Cancer Center Hangzhou 310003 China

**Keywords:** cancer therapy, cuproptosis, ferroptosis, nanomaterials, oxidative stress, reactive oxygen species, trace metal

## Abstract

Ferroptosis, as novel type of regulated cell death that has garnered widespread attention over the past decade, has witnessed the continuous discovery of an increasing number of regulatory mechanisms. Trace metal elements play a multifaceted and crucial role in oncology. Interestingly, it has been increasingly evident that these elements, such as copper, are involved in the regulation of iron accumulation, lipid peroxidation and antiferroptotic systems, suggesting the existence of “nonferrous” mechanisms in ferroptosis. In this review, a comprehensive overview of the composition and mechanism of ferroptosis is provided. The interaction between copper metabolism (including cuproptosis) and ferroptosis in cancer, as well as the roles of other trace metal elements (such as zinc, manganese, cobalt, and molybdenum) in ferroptosis are specifically focused. Furthermore, the applications of nanomaterials based on these metals in cancer therapy are also reviewed and potential strategies for co‐targeting ferroptosis and cuproptosis are explored. Nevertheless, in light of the intricate and ambiguous nature of these interactions, ongoing research is essential to further elucidate the “nonferrous” mechanisms of ferroptosis, thereby facilitating the development of novel therapeutic targets and approaches for cancer treatment.

## Graphical abstract

The induction of ferroptosis inhibits tumor growth, enhances anticancer efficacy, and overcomes drug resistance. Recent evidence shows nonferrous metal elements play a role in ferroptosis. This review focuses on how trace metals regulate ferroptosis processes like iron accumulation, lipid peroxidation, and antioxidant defense. Recent advances in using metal‐based nanomaterials to target ferroptosis in cancer therapy are also covered.

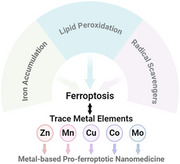

## 1 Introduction

Metal elements, especially those that belong to the “trace elements”, exert significant and essential regulatory function on various cellular biological processes.^[^ ^1^ ^]^ However, when their homeostasis is imbalanced, it can also stir up deleterious effects such as oxidative stress and even “metalloptosis”, the term proposed recently.^[^ ^2^ ^]^ Ferroptosis, one of the manifestations of dysregulated iron metabolism and initially conceptualized in 2012, is a mode of regulated cell death (RCD) characterized by iron‐dependent lipid peroxidation and accumulation of reactive oxygen radicals generated by Fenton reaction.^[^ ^3^ ^]^ It has been linked with many pathological conditions, including kidney diseases, neurodegenerative diseases, ischemic damage, cardiomyopathy, and of course, cancers. Although ferroptosis is regulated by multiple cellular metabolic pathways, there are mainly three key components: 1) the accumulation of iron, 2) the lipid peroxidation, and 3) the overwhelmed antioxidant defense system. Considering that many biological behaviors of cancer (such as in DNA repair, mitochondrial respiration, signal transduction, and regulation of gene expression) present in an iron‐dependent manner to better facilitate tumor cell survival and proliferation, iterative attempts have been made to target ferroptosis as a vulnerability to surmount cancers and have achieved promising outcomes.^[^ ^3c,d^ ^]^

Due to the similarities in the metabolism of certain metal elements within cells, as well as the complex interplay between them,^[^ ^4^ ^]^ it can be speculated that the homeostasis of other metals may also affect ferroptosis. Indeed, some studies across diverse contexts have elucidated the mechanisms of ferroptosis involving nonferrous elements.^[^ ^4b^ ^]^ Among them, copper may exhibit the strongest association with ferroptosis, not only owing to its multiple critical physiological functions but also its redox properties that are analogous to those of iron.^[^ ^5^ ^]^ Specifically, the crosstalk between copper metabolism and ferroptosis has been documented in chronic kidney disease, osteoarthritis, and nonalcoholic fatty liver disease (NAFLD).^[^ ^6^ ^]^ Furthermore, cuproptosis, a recently identified metal‐driven mechanism of cell death proposed in 2022, can be broadly considered as a component of copper metabolism that interacts with ferroptosis across various diseases, including cancer.^[^ ^7^ ^]^ These studies all indicate that copper is extensively involved in the physiological metabolism of iron and ferroptosis. Regarding other essential metal elements, including zinc,^[^ ^8^ ^]^ manganese,^[^ ^9^ ^]^ cobalt,^[^ ^10^ ^]^ and molybdenum,^[^ ^11^ ^]^ they exert varying degrees of influence on ferroptosis within specific disease contexts. This indicates the presence of a significantly more intricate metal regulatory network than previously anticipated. However, there is an absence of systematic reviews in oncology addressing the interactions between metal elements and ferroptosis. Further studies and reviews will enhance our understanding of cancer ferroptosis, aiding the development of potential treatment strategies to promote ferroptosis in refractory cancers.

In this review, we summarize the molecular mechanisms underlying ferroptosis, elucidate the physiological metabolism and homeostatic alterations of copper and explore its relationship with ferroptosis. We also examined potential avenues of evidence regarding the regulation of cancer ferroptosis by other trace metal elements (zinc, manganese, cobalt, molybdenum), with the objective of providing comprehensive guidance for clinical translation.

## 2 Overview of the Mechanisms of Ferroptosis

**Figure 1:**
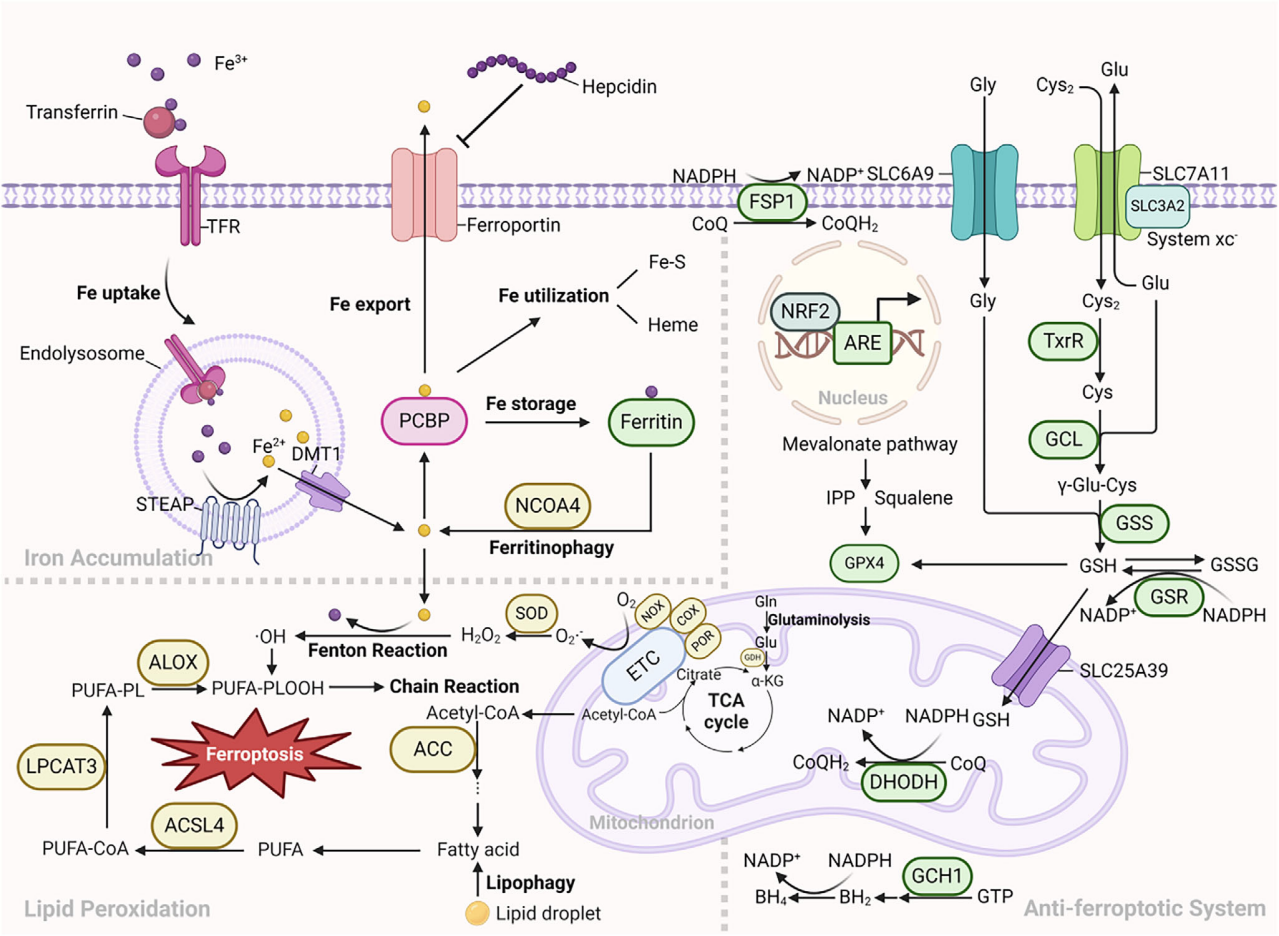
The overview of ferroptosis. Ferroptosis mainly presents three major features: 1) Iron accumulation: Iron homeostasis encompasses the processes of iron uptake, utilization, storage and export. The iron accumulation resulting from the dysregulation of any of these parts may trigger the Fenton reaction and serve as the initiating factor for ferroptosis. 2) Lipid peroxidation: Polyunsaturated fatty acids (PUFAs) enhance the generation of ROS. The enzymes that facilitate the synthesis of PUFAs and those associated with the mitochondrial respiratory chain are capable of promoting lipid peroxidation. 3) Imbalance of the antiferroptotic system: The system xc^−^‐GSH‐GPX4 axis serves as the primary pathway for detoxifying lipid peroxides into corresponding alcohols. Other antioxidants, such as reduced coenzyme Q (CoQH_2_) and tetrahydrobiopterin (BH_4_), can compensate for the lack of function in the GPX4 system. The NRF2‐ARE (antioxidant response element) axis is accountable for the overall control of oxidative stress in cells. The yellow patterns in the figure represent proferroptotic molecules, while the green patterns represent antiferroptotic molecules.

Ferroptosis, initially identified as a nonapoptotic cell death dependent on iron rather than other metals, is characterized by the accumulation of intracellular iron, lipid peroxidation and crash of the antioxidant defense system.^[^ ^3^, ^12^ ^]^ Erastin, the first reported potent inducer of ferroptosis, inhibits cystine uptake by blocking the system xc^−^ (the cystine/glutamate antiporter which consists of two subunits Solute Carrier Family 7 Member 11 (SLC7A11) and Solute Carrier Family 3 Member 2 (SLC3A2)), thereby impacting the subsequent synthesis of glutathione (GSH), one of the most critical and abundant endogenous antioxidants within cells. Consistently, RAS‐selective‐lethal‐3 (RSL3), upon binding, inactivates glutathione peroxidase 4 (GPX4, the indispensible enzyme for lipid hydroperoxides detoxification) and then promotes ferroptosis.^[^ ^13^ ^]^ Instead, ferrostatin‐1, one of the synthetic radical‐trapping antioxidants (RTAs), scavenges lipid peroxides and counters ferroptosis. Consequently, the fundamental mechanism of ferroptosis involves the collapse of the antioxidant defense system due to oxidative damage induced by iron catalysis, which contributes to lipid peroxidation. Below is the summary of the core mechanisms of ferroptosis and will be elaborated in the following sections (**Figure** **1**).

### 2.1 Iron Accumulation

In accordance with many instances of metal overload, excessive iron has long been shown to be closely associated with oxidative stress and widely studied in brain diseases and aging.^[^ ^14^ ^]^ Given that many cancers have an increased demand for iron (ferroplasia, a kind of metalloplasia), ferroptosis or iron toxicity mediated by excess iron accumulation therefore becomes a vulnerability of cancer cells.^[^ ^15^ ^]^ The metabolism of iron encompasses the transport of iron in plasma, the uptake of iron by cells, its translocation, utilization and storage within cells, as well as the export from cells.^[^ ^3a^ ^]^ The cellular and systemic iron homeostasis can also be regulated by iron‐regulatory proteins (IRPs) and the hepcidin‐ferroportin (FPN) axis. Hence, any misstep is likely to result in the accumulation of iron, and the influence of copper metabolism will also be manifested during this process.

### 2.2 Lipid Peroxidation

Owing to the widely recognized high metabolic traits, cancer cells possess higher levels of reactive oxygen species (ROS) compared to normal cells.^[^ ^16^ ^]^ The massive uptake of extracellular substances, aberrant mitochondrial respiration, and the stress triggered by immune system attacks can all give rise to the generation of a considerable amount of ROS. Subsequently, the iron‐catalyzed Fenton reaction can produce large amounts of hydroxyl radicals, which are the most active substances within cells, leading to a chain reaction of phospholipid peroxidation, ultimately destroying the integrity of the membrane. Some oxidative enzymes, such as NADPH oxidase (NOX), lipoxygenase (LOX), and cytochrome P450 oxidoreductase (POR), play a crucial role in the production of ROS.^[^ ^3b,c^ ^]^ For the synthesis of biological membranes and signaling molecules, the fatty acid metabolism of cancer cells undergoes numerous alterations.^[^ ^17^ ^]^ Against this backdrop, adequate fatty acid supply becomes another decisive contributor to lipid peroxidation. Furthermore, other metabolic pathways in cancer cells, such as carbohydrate and amino acid metabolism, can likewise influence lipid metabolism or facilitate the generation of ROS.

### 2.3 Antioxidant Defense System

As previously mentioned, ferroptosis can be potentized by antioxidant inhibitors and counteracted by RTAs. The most important antioxidant pathway against lipid peroxidation within cells is the system xc^−^‐GSH‐GPX4 axis. Other endogenous defense mechanisms include the ferroptosis suppressor protein 1 (FSP1)‐ubiquinol (CoQH_2_, the reduced form of ubiquinone system),^[^ ^18^ ^]^ the dihydroorotate dehydrogenase (DHODH)‐CoQH_2_ system, the sterol C5‐desaturase (SC5D)‐7‐dehydrocholesterol (7‐DHC) system,^[^ ^19^ ^]^ the GTP cyclohydrolase 1 (GCH1)‐tetrahydrobiopterin (BH4) system,^[^ ^20^ ^]^ and the endosomal sorting complexes required for transport (ESCRT)‐III‐dependent membrane repair system.^[^ ^21^ ^]^ These antioxidant effect molecules are largely governed by a network of antioxidant transcription factors centered around Nuclear Factor Erythroid 2‐Related Factor 2 (NRF2, encoded by NFE2L2).^[^ ^22^ ^]^ Incidentally, some natural exogenous antioxidants, such as selenium, vitamin E and vitamin K, can also exert a function of attenuating ferroptosis.^[^ ^23^ ^]^ Given the importance of the antioxidant system, the interaction between nonferrous metal elements and antioxidants may affect ferroptosis under certain conditions.

## 3 Crosstalk of Copper Metabolism and Ferroptosis in Cancer

### 3.1 The Physiological Metabolism of Copper

**Figure 2:**
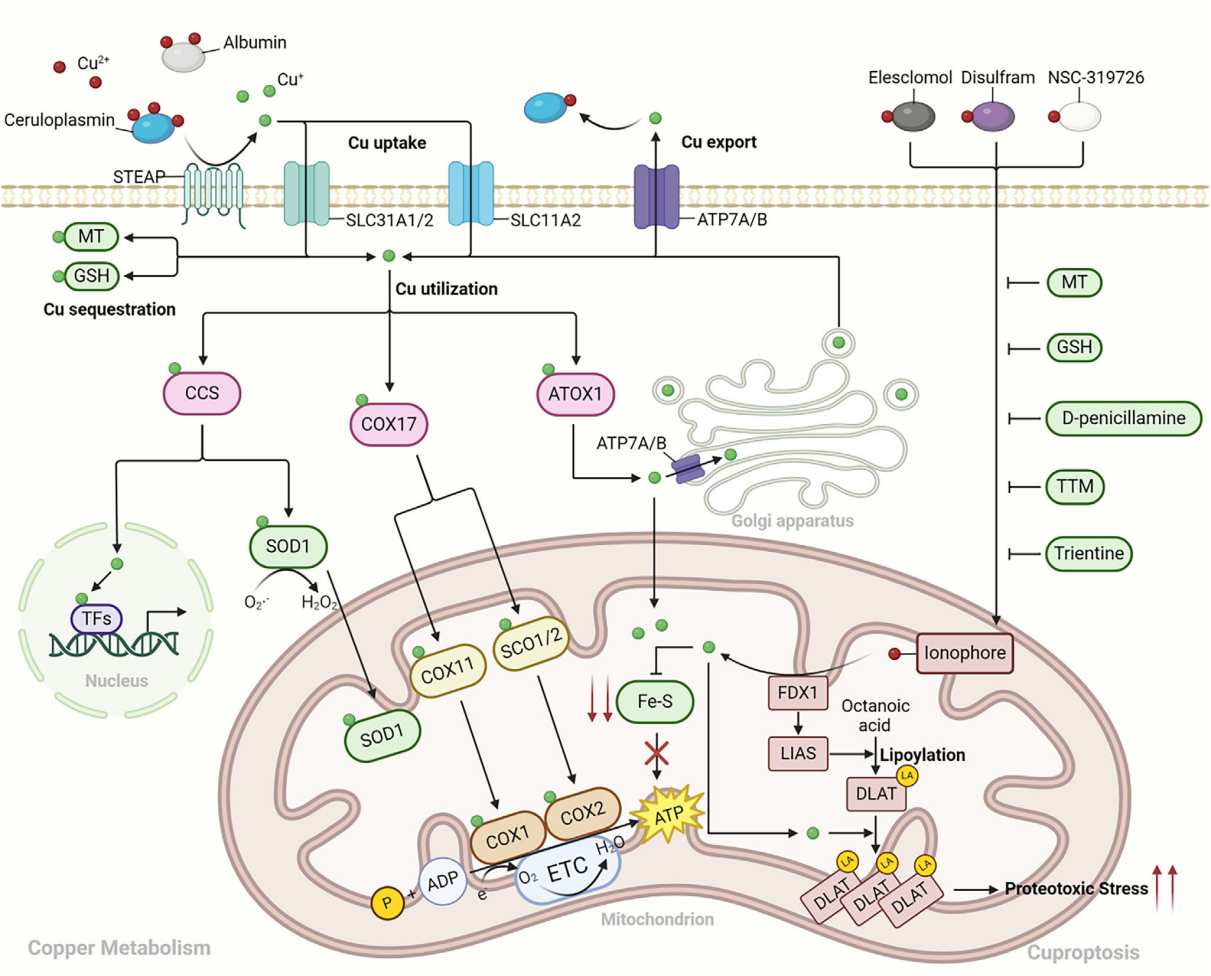
Copper metabolism and cuproptosis. The Cu^2+^ transported by ceruloplasmin is predominantly reduced to Cu^+^ at the cell membrane and subsequently enters the intracellular compartment. Within the cytoplasm, Cu^+^ combines with multiple copper chaperone proteins and is conveyed to nuclei, cytoplasmic copper enzymes, mitochondria, and the Golgi apparatus for utilization. GSH and metallothionein are accountable for sequestering copper to abrogate the toxic effects of excessive Cu^+^. Cu^+^ is mainly extruded from the cell via the Golgi apparatus‐vesicular system with the assistance of ATP7A/B. Copper ionophores can directly transport Cu^2+^ into cells. Cuproptosis denotes a mode of regulated cell death that is triggered by Cu^+^‐induced lipoylation of mitochondrial enzymes like Dihydrolipoamide S‐acetyltransferase (DLAT), thereby leading to a decrease in iron‐sulfur clusters and overwhelming proteotoxic stress. Copper chelators such as D‐penicillamine, tetrathiomolybdate (TTM) and trientine can alleviate cuproptosis. The brown and pale pinkish‐grey patterns in the figure represent procuproptotic molecules, while the green patterns represent anticuproptotic molecules.

On average, one adult intake 0.8 mg of copper daily through diet. After absorption by the small intestine, it is mainly transported to the liver by ceruloplasmin and then re‐secreted and distributed throughout the body.^[^ ^24^ ^]^ As a transition metal element, similar to iron, copper possesses two ionic forms: cuprous ion (Cu⁺), which demonstrates both oxidizing and reducing capabilities; and cupric ion (Cu^2^⁺), which exhibits a certain degree of oxidizing nature. Hence, the valence conversions of copper and iron within cells, to a certain extent, reflect and affect the redox status of cells, and are closely associated with oxidative stress, mitochondrial function and cell death. Notably, resembling iron, copper is capable of binding to regulatory proteins of various signaling pathways and influencing their activities, thereby participating in processes such as energy conversion, lipid metabolism, cell proliferation and autophagy, which promotes tumor progression, angiogenesis, and metastasis.^[^ ^7^, ^15^, ^24^ ^]^ This is the so‐called concept of cuproplasia. The process of copper metabolism is exhibited below (**Figure** **2**).

#### 3.1.1 Cellular Uptake of Copper

In the absence of copper ionophores, copper is predominantly transported into the cell through Solute Carrier Family 31 Member 1/2 (SLC31A1/SLC31A2, also known as Copper Transporter 1/2 (CTR1/CTR2)) in the form of Cu⁺.^[^ ^25^ ^]^ Consequently, the Cu^2+^ bound to ceruloplasmin, representing 95% of the copper ions in the blood, must initially be reduced by the metalloreductase Six Transmembrane Epithelial Antigen of the Prostate (STEAP).^[^ ^26^ ^]^ Whereas the affinity of SLC31A2/CTR2 toward Cu⁺ is markedly lower than that of SLC31A1/CTR1.^[^ ^27^ ^]^ Furthermore, Cu⁺ can also enter cells via Divalent Metal Transporter 1 (DMT1, encoded by the SLC11A2 gene). DMT1 is predominantly distributed on the endolysosomal membranes as well as the cell membrane and plays a crucial role in the absorption of divalent iron.^[^ ^7^, ^24^ ^]^

#### 3.1.2 Copper Distribution and Utilization

Studies have indicated that cells with vigorous mitochondrial energy metabolism exhibit a greater reliance on Cu^+^.^[^ ^15^ ^]^ Therefore, it is not surprising that the organelle where Cu^+^ is most abundantly distributed is the mitochondrion. Cu^+^ is recruited by the Cytochrome C Oxidase Copper Chaperone (COX17) and is eventually transferred to mitochondrion for incorporation into the COX1 and COX2 (encoded by *MT‐CO1/2*, *Mitochondrially Encoded Cytochrome C Oxidase 1/2*) in the inner mitochondrial membrane for electron transport and energy production. During the transfer of Cu^⁺^ to COX1/2, the assistance of Cytochrome C Oxidase Subunit 11 (COX11) and Synthesis of Cytochrome C Oxidase 1/2 (SCO1/2) is respectively required.^[^ ^28^ ^]^ In the cytosol, the Copper Chaperone for Superoxide Dismutase (CCS) transfers Cu^+^ to superoxide dismutase 1 (SOD1), thereby facilitating its capacity to convert harmful superoxide free radicals (O_2_^·−^) to molecular oxygen or hydrogen peroxide. CCS can also transport Cu⁺ to the nucleus, where Cu^+^ activates specific transcription factors and thereby regulating gene expression.^[^ ^29^ ^]^ Another subcellular compartment in which Cu⁺ is predominantly distributed is the Golgi apparatus, where it collaborates with chaperones to facilitate protein processing. Specifically, Cu⁺ binds with Antioxidant 1 Copper Chaperone (ATOX1) in the cytoplasm, and subsequently is conveyed by the latter to ATPase Copper Transporting A/B (ATP7A/B) on the Golgi membrane and enters therein.^[^ ^5^ ^] [^ ^30^ ^]^

#### 3.1.3 Cellular Export of Copper

ATP7A/B located on cytomembrane is involved in the export of copper out of the cells. The Cu⁺ that enters the bloodstream is promptly oxidized by ceruloplasmin to Cu^2+^ for transportation. The surplus copper in the organism is chiefly excreted via bile and feces. ATP7A/B can also be translocated from the Golgi apparatus to the cytomembrane through the vesicles produced concomitantly, so as to facilitate the efflux of excessive copper and maintain the appropriate level of copper ions within the cells.^[^ ^24^ ^]^

#### 3.1.4 Copper Sequestration

Owing to the similar redox‐active hallmark, copper, like iron, is capable of generating a substantial amount of detrimental ROS via the Fenton reaction.^[^ ^31^ ^]^ Consequently, cells have evolved two major copper sequestration mechanisms to restrain the chemical reactivity of copper. In terms of eliminating labile Cu⁺ within cells, the most significant substance remains GSH.^[^ ^32^ ^]^ It restricts Cu⁺‐mediated signal transduction and the toxicity induced by oxidative stress through sequestering copper. Another natural chelator for Cu^+^ is the sulfur‐containing protein metallothionein (MT).^[^ ^33^ ^]^ Proteins of this family are rich in cysteine residues, endowing them with an excellent capacity to bind and detoxify heavy metal ions. Therefore, factors regulating GSH and metallothionein will also have an impact on copper homeostasis.

### 3.2 Copper Metabolism and Iron Accumulation in Ferroptosis of Cancer

#### 3.2.1 Copper Metabolism and Iron Uptake/Export

The major copper transport protein in plasma, ceruloplasmin, is a significant ferroxidase, capable of oxidizing Fe^2+^ to Fe^3+^ and facilitating the binding of Fe^3+^ to transferrin. This oxidative function likewise holds pivotal importance within the cell. Ceruloplasmin is indispensable in the iron cycle, and its deficiency is likely to influence the cellular uptake and export of iron, thereby disrupting the ferroptosis.^[^ ^7^, ^24^, ^34^ ^]^ However, in the realm of cancer biology, the prevailing perspective holds that ceruloplasmin plays a certain role in suppressing ferroptosis via facilitating the oxidation of intracellular free Fe^2+^ and enhancing iron export, yet it remains unclear whether its promotion of iron transport and absorption would further promote ferroptosis. For instance, in hepatocellular carcinoma (HCC), the increase in intracellular copper levels induced by ionizing radiation promotes the transcriptional activity of Hypoxia Inducible Factor 1 Subunit Alpha (HIF1A), thereby elevating the expression levels of ceruloplasmin and SLC7A11 within cells. This process reflects the antiferroptotic role of copper/ceruloplasmin and can be inhibited by Copper Metabolism MURR1 Domain 10 (COMMD10).^[^ ^35^ ^]^ Ceruloplasmin can also interact with FPN to promote the excretion of Fe^2+^ in HCC.^[^ ^36^ ^]^ It is notable that, in fibrosarcoma and endometrial cancer, the levels of ceruloplasmin in cancer cells are respectively modulated by vesicles secreted by tumor‐associated macrophages and long noncoding RNA (lncRNA).^[^ ^37^ ^]^ In light of the fact that direct interference with ceruloplasmin might bring about severe side effects, these findings indicate that targeting cancer‐specific regulators of ceruloplasmin may reverse therapeutic resistance or retard tumor progression.

Since the binding of transferrin receptor (TFR) to transferrin is the principal route for cells to uptake iron (Fe^3+^), the regulation of transferrin receptor activity may have an impact on the process of ferroptosis.^[^ ^3a^ ^]^ In breast cancer, Mediator of Cell Motility 1 (MEMO1) is capable of binding to transferrin receptor (TFR) and being involved in the regulation of ferroptosis.^[^ ^38^ ^]^ Additionally, a study has indicated that Cu^+^ could bind to MEMO1.^[^ ^39^ ^]^ We hypothesize that Cu^+^ may influence the level of TFR through binding to MEMO1 to affect ferroptosis. However, there is a lack of evidence as to whether this process eventually influences ferroptosis regulated by TFR.^[^ ^38^, ^39^ ^]^ Following the entry of the TFR‐transferrin‐Fe^3+^ complex into the endolysosome via endocytosis, reduction to Fe^2+^ by the metalloreductase STEAP is required, after which Fe^2+^ is transported to the cytoplasm by DMT1 to replenish the labile iron pool. Therefore, inhibition of DMT1 may result in ferroptosis induced by iron overload in the endolysosome.^[^ ^40^ ^]^ However, a liposome reported recently boosted DMT1 on endolysosomal membrane, thereby significantly facilitating the influx of Fe^2+^ into the cytoplasm and subsequently inducing ferroptosis in different cancer cells.^[^ ^41^ ^]^ On the surface of the cell membrane, Fe^3+^ is reduced to Fe^2+^ by ferroreductase Cytochrome B Reductase 1, also functions as a cupric reductase (CYBRD1), and Fe^2+^ can be transported into the cytoplasm via DMT1.^[^ ^3^, ^42^ ^]^ The elevated expression of DMT1 is regarded as the mechanism through which temozolomide induces ferroptosis in glioblastoma cells.^[^ ^43^ ^]^ As DMT1 serves as an essential channel for copper transport as well, we speculate that the copper level might have an impact on the iron transport capacity of DMT1 and exert an influence on ferroptosis. Nevertheless, on the whole, the regulatory mechanisms of copper on non‐TFR iron transporters such as DMT1, ZIP8 (encoded by *SLC39A8*), and ZIP14 (encoded by *SLC39A14*) remain ambiguous where more research is needed.

**Figure 3:**
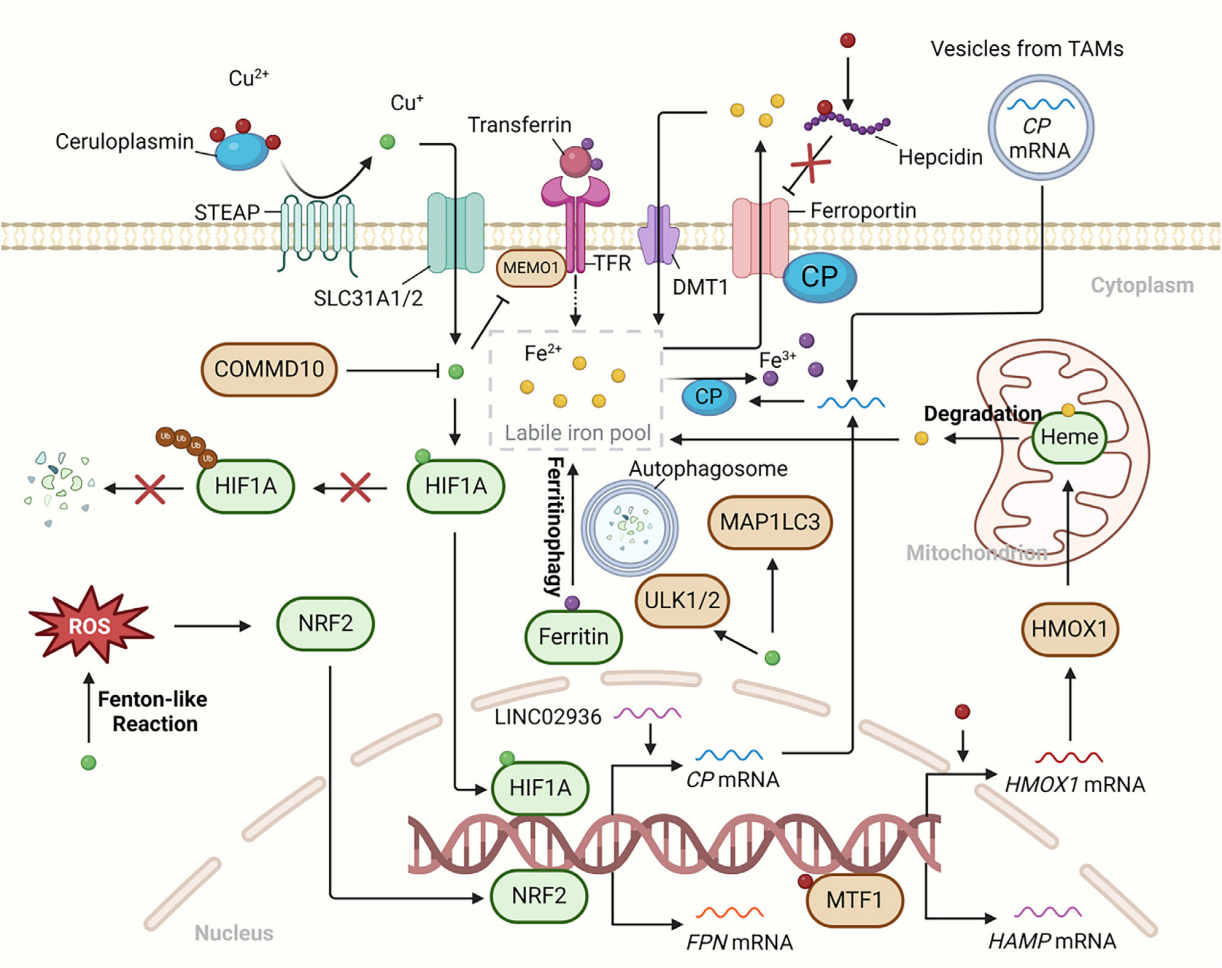
Copper metabolism and iron accumulation. Copper can modulate the functions of transcription factors such as HIF1A, NRF2, and Metal Regulatory Transcription Factor 1 (MTF1), thereby altering the expression of genes related to iron metabolism within cells and exerting a promoting or inhibitory effect on ferroptosis. Copper or proteins associated with copper metabolism can likewise directly modulate iron uptake, utilization and export, thereby altering the content of the labile iron pool. Furthermore, copper might potentially promote the ferritinophagy and release the stored iron. The brown patterns in the figure represent proferroptotic molecules, while the green patterns represent antiferroptotic molecules.

The efflux of iron is primarily mediated by the ferroportin (FPN, encoded by the *SLC40A1* gene) – hepcidin (encoded by the *Hepcidin Antimicrobial Peptide (HAMP)* gene) axis, among which hepcidin is a cysteine‐rich antibacterial polypeptide capable of binding to FPN and facilitating its degradation.^[^ ^3^, ^44^ ^]^ One study indicated that in macrophages, the oxidative stress triggered by short‐term copper loading activated NRF2 and then facilitated the expression of FPN.^[^ ^45^ ^]^ However, the regulatory effect of copper on FPN in tumor cells has not been verified. Notably, the induction of hepcidin expression by copper and other metals has been reported in the Huh7 HCC cell line,^[^ ^46^ ^]^ which may promote ferroptosis by hindering iron export. Cu^2+^ also possesses the ability to combine with hepcidin in plasma. However, due to its relatively low affinity, it may not have a significant effect on iron homeostasis and ferroptosis^[^ ^47^ ^]^ (**Figure** **3**).

#### 3.2.2 Copper Metabolism and Iron storage

The predominant form for iron storage within cells is ferritin that is constituted by heavy chains and light chains (encoded by *FTH1* and *FTL*, respectively), among which the heavy chain is capable of oxidizing Fe^2+^ to Fe^3+^. The iron bound to ferritin can be reclaimed to the labile iron pool through the ferritinophagy mostly mediated by the cargo protein Nuclear Receptor Coactivator 4 (NCOA4), which facilitates ferroptosis and has demonstrated potential clinical application value in cancer treatment.^[^ ^48^ ^]^ Since the NCOA4 bound to ferritin ultimately be delivered to the autophagosome by binding to autophagy‐related (ATG) proteins such as Microtubule Associated Protein 1 Light Chain 3 (MAP1LC3), factors influencing the autophagy process will impact ferritinophagy. This aligns with the autophagy‐dependent properties of ferroptosis.^[^ ^49^ ^]^ Mechanistically, the influence of copper on ferritinophagy may be realized through various manners. Research indicated that in lung adenocarcinoma (LUAD), the combination of copper and Unc‐51 Like Autophagy Activating Kinase 1/2 (ULK1/2) was a requisite for the formation of autophagosomes, and the inhibition of the copper transporter CTR1 markedly suppresses autophagy.^[^ ^50^ ^]^ We speculate that the existence of copper in LUAD can enhance ferroptosis by promoting ferritinophagy. Yet, contrary conclusions were reached in pancreatic cancer.^[^ ^51^ ^]^ This spurs researchers to undertake context‐specific studies in diverse types of cancer. Furthermore, excessive copper regulated the autophagy flux by directly modulating the expression of ATG proteins, such as ATG‐5, Beclin1, p62, and MAP1LC3.^[^ ^52^ ^]^ The oxidative stress caused by copper could also promote autophagy through activating the AMP‐Activated Protein Kinase (AMPK)‐ Mammalian Target of Rapamycin (mTOR) pathway.^[^ ^53^ ^]^ Despite the lack of evidence for the direct interaction between copper and NCOA4, it is conceivable that exploiting the regulatory characteristics of copper on autophagy might be an effective strategy for potentiating ferroptosis to benefit cancer treatment.

Incidentally, for the stable coordination with Fe^2+^ and the transportation thereof to the ferritin storage or other cellular compartments for further utilization, Poly (RC) Binding Protein (PCBP) and GSH are indispensable.^[^ ^54^ ^]^ However, as of now, no studies have indicated the association between copper metabolism and PCBP (Figure 3).

#### 3.2.3 Copper Metabolism and Iron Utilization

A significant pathway for iron utilization is the synthesis of heme within cellular mitochondria. Heme, an iron porphyrin compound, serves as a cofactor for various proteins, including hemoglobin, cytochromes, peroxidases and catalase.^[^ ^55^ ^]^ Consequently, iron plays a vital role in sustaining the functions of numerous proteins. Nevertheless, analogous to iron, heme is also toxic when in excess.^[^ ^55^ ^]^ Copper has long been found to regulate heme homeostasis and *Heme Oxygenase 1* (*HMOX1*, encoding an important enzyme for heme metabolism and possesses multiple functions in cancer and is usually stress‐induced), a copper‐responsive gene.^[^ ^56^ ^]^ Recent investigations have demonstrated that both copper ionophore disulfiram (DSF)‐Cu and copper nano‐delivery drugs/nanozymes could induce the expression of HMOX1. This procedure facilitated the degradation of heme and the liberation of free iron, thereby promoting ferroptosis in tumor cells and generating a remarkable synergistic effect in conjunction with other antitumor therapies.^[^ ^56^, ^57^ ^]^ Therefore, heme homeostasis is also an interactive hub for copper metabolism and ferroptosis.

The synthesis of iron‐sulfur clusters (ISCs) represents another key utilization pathway for iron. ISCs are primarily localized in the mitochondria and are among the most conserved and ubiquitously present biomolecules, playing essential roles in electron transfer and metabolism regulation.^[^ ^58^ ^]^ A deficiency in ISCs leads to increased iron load and promotes ferroptosis.^[^ ^59^ ^]^ In cuproptosis, Cu⁺ binds to lipoylated proteins in the tricarboxylic acid (TCA) cycle, leading to their aggregation and degradation in ISCs, which induces proteotoxic stress and ultimately results in cell death.^[^ ^7a^ ^]^ Thus, the reduction of ISCs may act as a convergence point between cuproptosis and ferroptosis and will be detailed further below. However, current studies mainly concentrate on the alterations of ISCs in the context of cuproptosis, and very little is known about whether the regulation of ISCs by copper directly affects ferroptosis in cancers (Figure 3).

### 3.3 Copper Metabolism and Lipid Peroxidation in Ferroptosis of Cancer

#### 3.3.1 Copper Metabolism and Biosynthesis of Polyunsaturated Fatty Acids

Due to the fact that polyunsaturated fatty acids (PUFAs), a kind of fatty acids that contain more than one unsaturated carbon bond, are most vulnerable to ROS and prone to lipid peroxidation, their synthesis and supplementation constitute significant initiating factors for ferroptosis.^[^ ^3^, ^60^ ^]^ Hence, the interaction between copper and the metabolism of PUFAs might influence the susceptibility of cells to ferroptosis. The primary source of fatty acids is de novo synthesis, utilizing acetyl‐CoA derived from glucose and amino acid metabolism. Acetyl‐CoA is converted to malonyl‐CoA catalyzed by acetyl‐CoA carboxylase (ACC), representing the rate‐limiting step in the process. Copper can affect the expression level of ACC in other species, which may also apply to human cells.^[^ ^61^ ^]^ Given that lipid droplets are essential for lipid storage, they also serve as the primary source of raw materials (free fatty acids) for synthesizing PUFAs.^[^ ^62^ ^]^ Studies have confirmed that the biogenesis of lipid droplets and the intracellular accumulation of copper are positively correlated to a certain extent.^[^ ^63^ ^]^ Lipophagy, an innovative lipid droplet metabolic modality, has the capacity to boost intracellular free fatty acid concentrations and expedite PUFAs biosynthesis, which fuels the ferroptosis.^[^ ^3^, ^64^ ^]^ Notwithstanding the vigorous lipid metabolism, it remains undetermined whether copper influences the lipophagy of lipid droplets in tumor cells. Additionally, copper can also enhance lipolysis of triglyceride (TG) through the c‐AMP/Phosphodiesterase 3B (PDE3B) axis to release free fatty acids.^[^ ^65^ ^]^

**Figure 4:**
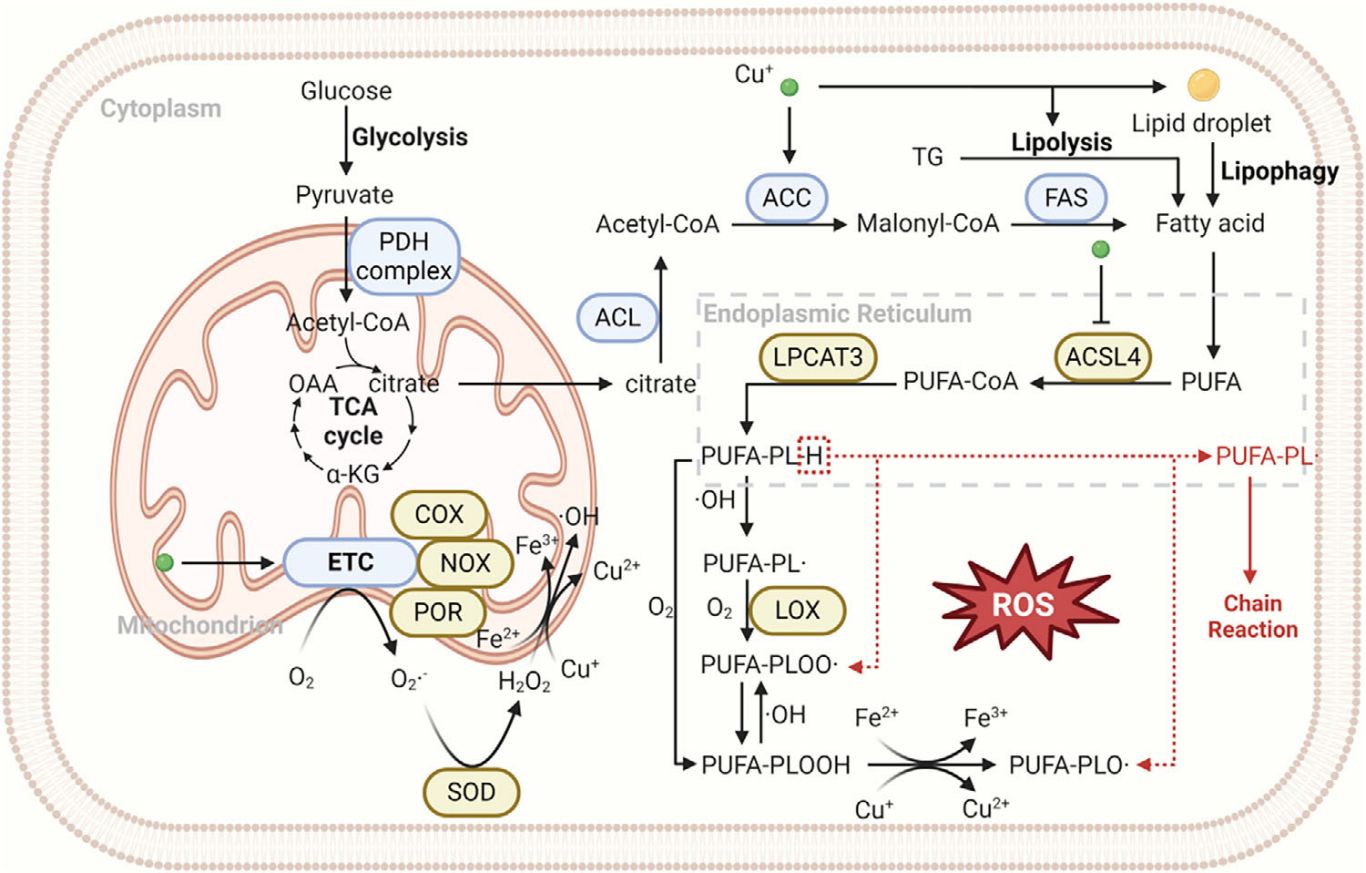
Copper metabolism and lipid peroxidation. Regarding the synthesis of PUFAs, Cu^+^ can supply raw material free fatty acids by facilitating lipophagy of lipid droplets and lipolysis of triglycerides (TG). The current research on the effect of Cu^+^ on the enzymes for PUFA synthesis is scarce. In terms of the generation of ROS, given that Cu^+^ is a cofactor of numerous mitochondrial respiratory chain enzymes, it is capable of exerting an influence on oxidative phosphorylation and the generation of ROS. Moreover, in view of the fact that Cu^+^ possesses redox properties similar to Fe^2+^, it can undergo a Fenton‐like reaction with H_2_O_2_ and react with lipid peroxides to generate a greater number of free radicals. The yellow patterns in the figure represent proferroptotic molecules.

As lipid peroxidation in ferroptosis usually takes place on biomembranes, the substrate of the chain reaction is actually polyunsaturated fatty acid‐containing phospholipid (PUFA‐PL). Therefore, the two crucial enzymes for the synthesis of PUFA‐PL, namely the phospholipid remodeling enzymes, namely acyl‐CoA synthetase long‐chain family member 4 (ACSL4) and lysophosphatidylcholine acyltransferase 3 (LPCAT3), are typically highly expressed in several tumors and are intimately associated with ferroptosis susceptibility, thereby emerging as potential molecules for targeting cancer ferroptosis, which has been reviewed elsewhere.^[^ ^3^, ^66^ ^]^ Under intermittent hypoxia (IH) conditions, copper deficiency conspicuously upregulated the expression of ACSL4 in HepG2 cell and facilitated the transformation of PUFA to PUFA‐CoA, an intermediate for the synthesis of PUFA‐PL. However, knockdown of ACSL4 eradicated the lipid accumulation and ferroptosis induced by copper deficiency.^[^ ^67^ ^]^ This indicates that an appropriate amount of copper exhibits an antiferroptotic role under certain conditions. LPCAT3 catalyzes the transfer of fatty acyl chains from fatty acyl‐CoA to 1‐acyl lysophosphatidylcholine to form various types of phospholipids, but it is not clear whether copper affects the expression level or activity of LPCAT3^[^ ^66b^ ^]^ (**Figure** **4**).

#### 3.3.2 Copper Metabolism and ROS‐Producing Enzymes

The lipid peroxidation induced by ROS is contingent upon lipoxygenase (LOX, a class of nonheme iron‐containing enzymes that can oxidize PUFA‐PL to form phospholipid hydroperoxides (PUFA‐PLOOHs)), with distinct lipoxygenases playing distinct roles in the ferroptosis of tumor cells.^[^ ^3c^ ^]^ PUFA‐PLOOH is both the principal initiator and the product of peroxidative chain reactions.^[^ ^68^ ^]^ The latest research indicates that arachidonate 12/15‐lipoxygenase (ALOX12/15) in sheep are closely related to the regulation of copper homeostasis, which may facilitate further research in human cells.^[^ ^69^ ^]^ Other enzymes responsible for generating ROS during ferroptosis mainly consist of electron transport chain proteins, such as NOX, POR, and Cytochrome B5 Reductase 1 (CYB5R1).^[^ ^70^ ^]^ A newly developed copper‐based nanoenzyme exhibited stronger activity of NOX and other oxidases, which boosted the production of ROS in cancer cells.^[^ ^71^ ^]^ Similarly, while the interplay between copper or other metals and ROS‐producing enzymes remains not fully elucidated, the incorporation of metal‐based nanomaterials (including nanoenzymes) can alter intracellular redox homeostasis and facilitate the generation of an excess of ROS. The integration of metal complex‐driven ferroptosis with the existing therapeutic methods that induce other types of cell death (e.g., immunogenic cell death), such as immunotherapy, is anticipated to constitute a novel clinical anticancer paradigm.^[^ ^72^ ^]^ **Table** **1** presents the metal‐based nanomaterials capable of inducing ferroptosis and their applications (Table 1) (Figure 4). Despite the numerous reports from preclinical studies, as of now, no satisfactory progress has been made in the clinical trial for the ferroptosis‐inducing metal nanomaterials used in anticancer therapy, let alone their long‐term therapeutic efficacy. Concerns over the safety and efficacy of these materials may be a huge obstacle to their translation. Although the side effects of them in animals have been rarely reported in the existing preclinical studies, the influence of metal nanomaterials on human health and the environment must draw adequate attention. For the human body, in the short term, these nanomaterials may trigger acute toxic responses, allergic reactions and immune disorders; in the long term, the potential side effects encompass DNA or chromatin aberrations resulting from metal accumulation (with carcinogenic risks) as well as organ functional deterioration.^[^ ^73^ ^]^ Therefore, in preclinical studies, emphasis should also be placed on developing highly sensitive toxicity assessment and control techniques to facilitate the application of these metal complexes.

**Table 1:** Preclinical studies of metal‐based nanomaterials to induce ferroptosis in cancer cells.

Nanomaterials	Cancer Type (Cell Line)	Mechanisms of Inducing Ferroptosis	Refs.
*Copper‐based nanoparticles or nanoenzymes inducing ferroptosis*			
Copper‐cysteamine nanoparticle	CRC (HT115)	Synergized with microwave‐mediated photodynamic therapy (PDT) to deplete GPX4 and resulted in the accumulation of lipid peroxides and malondialdehyde (MDA).	[74]
Chlorin e6 @Cu Nanoparticle	GBM (U87MG)	Triggered a sonodynamic effect and depleted GSH.	[75]
Self‐assembled copper‐alanine nanoparticle loaded with glucose oxidase and cinnamaldehyde	Breast cancer (4T1)	By reducing Cu^2+^ to Cu^+^, GSH was consumed and generated large amounts of ROS, which killed tumor cells synergistically with immunotherapy.	[76]
DDC/Cu‐Fe encapsulated by albumin/lactoferrin nanoparticle	Glioma (GL261)	Inhibited GPX4‐GSH axis and promoted Fe accumulation as well as activated T‐cell immunity in tumor microenvironment.	[77]
Double layered hollow mesoporous cuprous oxide nanoparticle	Breast cancer (4T1, MCF‐7)	The Cu^+^ released by the carrier itself could induce ferroptosis in noncancer stem cells (CSCs) through a Fenton‐like reaction and GSH depletion.	[78]
Cu‐Ag alloy nanoparticle	Breast cancer (4T1)	Exerted a function similar to that of COX (cytochrome c oxidase) and generated a large quantity of ROS.	[79]
“Sea urchin‐like” copper sulfide nanoparticle	Breast cancer (4T1)	Combined with chemodynamic therapy (CDT) to target SLC7A11 to suppress the synthesis of GSH and the activity of GPX4; the Fenton‐like reaction involving Cu^2+^ directly generated ROS and promoted lipid peroxidation.	[80]
Doxorubicin‐loaded copper peroxide‐mesoporous silica nanoparticle	Breast cancer (4T1)	Self‐supplied H_2_O_2_ to produce ROS, synergizing with chemotherapy to kill tumor cells.	[81]
Sorafenib‐loaded copper peroxide nanoparticle	Breast cancer (4T1)	Sorafenib disrupted the system xc^−^, and the concurrently released Cu^2+^ was reduced to Cu^+^, which subsequently generated a considerable amount of ·OH via the Fenton reaction.	[82]
Copper chlorophyllin‐based carbon dot (Chl‐D CD)	LUAD (A549) GBM (U87)	Induced a Fenton‐like reaction and augmented photodynamic therapy (PDT).	[83]
Near‐infrared (NIR)‐responsive HCuS nanocomposite	Breast cancer (4T1)	Stimulated the secretion of IFN (interferon)‐γ to promote ACSL4‐mediated lipid peroxidation and GSH consumption caused by system xc^−^ inhibition.	[84]
RNP@Cu_2_O@SPF	CRC (HT29, SW480)	Cu^+^ triggered Fenton‐like reaction and depleted significant GSH; carrier‐delivered CRISPR‐Cas9 RNP inhibited ATP7A expression, exacerbating copper accumulation.	[85]
MitCuOHA nanozyme	Cervical cancer (Hela), HCC (HepG2), Breast cancer (4T1, MCF‐7)	Designed with cysteine oxidase‐like, glutathione oxidase‐like and peroxidase‐like activities to deplete cysteine and GSH, generating highly active hydroxyl radicals.	[86]
Cu_2_WS_4_ nanozyme	Breast cancer (4T1)	Regulated NRF2/HMOX1/GPX4 axis and decreasing GSH as a GSH oxidase, which sensitized cancer cells to radiotherapy and immunotherapy.	[57c]
Copper‐based metal‐organic framework nanoplatform	Breast cancer (4T1)	Promoted the generation of hydroxyl radicals through near‐infrared light irradiation and induced ferroptosis and immunogenic cell death.	[87]
Bimetallic copper and ruthenium (Cu@Ru) core‐shell nanoparticles	Breast cancer (4T1)	Consumed large amounts of GSH and caused lipid peroxidation.	[88]
Cu‐BTC@DDTC	Melanoma (B16F10)	Downregulated SLC7A11 and suppressed the GPX4 antiferroptotic signaling.	[89]
CuMnO@Fe3O4 core‐shell nanozyme	Breast cancer (4T1)	Depleted GSH and potentiated Fenton and Fenton‐like reactions, which increased intracellular ROS levels.	[90]
*Manganese‐based nanoparticles or nanoenzymes inducing ferroptosis*			
MnMoOx nanoparticle	CRC (CT26)	Depleted GSH and inhibited GPX4, which enhanced immunotherapy.	[91]
IFN‐γ/uMn‐LDH	Breast cancer (4T1)	Mn ions greatly contributed to GSH consumption and hydroxyl radical production; IFN‐γ suppressed the expression of SLC7A11.	[92]
Arginine‐rich manganese silicate nanobubble (AMSM)	HCC (Huh7)	The degradation of AMSM was accompanied by GSH depletion and subsequent GPX4 inactivation.	[93]
Polydopamine‐coated manganese sulfide (MnS) nanocluster	Lung cancer (LLC)	The Mn^2+^ released exhibited GSH oxidase and peroxidase‐like activity and the H_2_S inhibited mitochondrial respiration and ATP production.	[94]
PEGylated manganese‐zinc ferrite nanocrystal (PMZFN)	Prostate cancer (PC3)	Enhanced lipid peroxidation due to GPX4 suppression and amplified immunogenic cell death through cGAS‐STING stimulated IFN‐β.	[95]
Lpo@MnS‐GOx	Prostate cancer (PC3)	The glucose oxidase transformed glucose into gluconic acid and H_2_O_2_, the former promoted the degradation of MnS and Mn^2+^ launched the Fenton‐like reactions.	[96]
Sorafenib‐loaded manganese‐silica nanoparticle	HCC (HepG2)	Mn^2+^ had the capacity of consuming GSH and led to the inactivity of GPX4; meanwhile, sorafenib effectively inhibited the biogenesis of GSH, further disrupted the redox homeostasis.	[97, 98]
Mn^2+^‐Aloe‐Emodin nanoparticle	Breast cancer (4T1)	The Mn^2+^ facilitated the generation of ROS through Fenton‐like reactions and Aloe‐Emodin effectively inhibited the activity of NRF2.	[99]
Perfluorocarbon‐MnOx core–shell nanoparticle	Breast cancer (4T1)	The perfluorocarbon enhanced the Mn^2+^‐induced GSH exhaustion and lipid peroxidation, which synergized with O_2_ supply to surmount hypoxia‐mediated ferroptosis resistance.	[100]
PEG‐PDA@Mn nanoparticle	Gastric cancer (MFC)	Combined with photothermal therapy (PTT) to produce catastrophic ROS.	[101]
PtMn nanoparticle	Breast cancer (4T1) CRC (CT26)	Increased malondialdehyde concentration and ROS levels, also disrupted mitochondrial membrane potential.	[102]
Mesoporous polydopamine‐coated GOx‐Mn nanoreactor	Cervical cancer (Hela)	The MnO_2_ shell released Mn^2+^ that depleted GSH via Fenton‐like reactions and was consolidated by H_2_O_2_ catalyzed from Gox, which sensitized cells to PTT.	[103]
Mn‐doped graphene quantum dot	HCC (HepG2)	Strengthened the generation of ROS and its accumulation in lysosome that led to the dysregulation of its’ function.	[104]
Camptothecin and MnO_2_‐coated polydopamine nanoparticle	Lung cancer (LLC)	Mn^2+^ induced ROS production and camptothecin inhibited DNA topoisomerase, which linked ferroptosis and apoptosis.	[105]
MnOx‐coated Au nanoenzyme	Breast cancer (4T1)	Catalyzed the oxidation of NADPH and hindered the regeneration of GSH.	[106]
Gelatin‐coated manganese‐doped mesoporous silica nanoparticle	Pancreatic cancer (SW1990)	Mn^2+^ catalyzed H_2_O_2_ to O_2_ and exhausted GSH.	[107]
*Zinc‐based nanoparticles or nanoenzymes inducing ferroptosis*			
“Iron free” ZnO nanoparticle	Breast cancer (MB231) HCC (Hepa 1–6) Cervical cancer (Hela)	Disrupted intracellular iron metabolism involving iron uptake, storage and export; promoted the depletion of GSH and downregulation of GPX4, which boosted the ROS generation and lipid peroxidation.	[108]
Bio‐barcode ZnO nanoparticle	Lung cancer (A549)	Depleted GSH and silenced *EGR‐1 (Early Growth Response Protein 1)* mRNA by Zn^2+^‐dependent enzymes to counter drug resistance.	[109]
Porphyrin‐based zinc ferrite magnetic nanozyme	Cervical cancer (Hela) Breast cancer (MCF‐7)	Presented multienzyme‐like cascade activity to generate O_2_^−^ and subsequent free radicals and could also increased intracellular Fe levels.	[110]
ZnO nanoparticle	ALL (Nalm‐6, REH)	Increased Fe levels and *ACSL4*, *ALOX15* mRNA expressions; decreased *SLC7A11* and *GPX4* mRNA expressions.	[111]
Virus‐like silica ZnO nanoparticle	CRC (CT26, HCT116)	Consumed endogenous H_2_S, and resulted in the depletion of GSH.	[112]
Hydrazone ligand‐based zinc complex	HCC (HepG2), Lung cancer (A549), Breast cancer (4T1), Cervical cancer (Hela)	Under ultrasound irradiation (US), singlet oxygen (^1^O_2_) is generated to consume GSH and inactivate GPX4, thereby causing cellular sonotoxicity (sonodynamic therapy, SDT).	[113]
Zn/Pt dual‐site single‐atom superimposition‐augmented TiO_2_‐based sonosensitizer	Breast cancer (4T1)	Zn and Pt atoms could assist electron (e^−^) excitation and produced more sonoexcited holes (h^+^) under US stimuli so as to generate more ROS, overcoming the drawback of rapid recombination of electrons and holes.	[114]
Mn‐doped ZnO nanocluster with enhanced piezoelectric effect	Breast cancer (4T1)	US‐generated holes strongly oxidized the GSH to form GSSG, which deleted GSH and inactivated GPX4 and was enhanced by Mn^2+^/Mn^3+^.	[115]
*Cobalt‐based nanoparticles or nanoenzymes inducing ferroptosis*			
Clustered cobalt nanodot	Breast cancer (4T1, MCF‐7) Lung cancer (A549) Gastric cancer (Caco‐2)	Upregulated HMOX1 that significantly increased TFR while decreased FPN, resulting in Fe^2+^ accumulation within cells and radiotherapy sensitization.	[116]
Co‐MION nanozyme	GBM (U87)	Exhibited peroxidase‐like activity to produce toxic ROS.	[117]
Lactobionic acid modified cobalt coordination polymer‐coated peroxymonosulfate nanoparticle	HCC (HepG2)	Co^2+^ activated HSO_5_^−^ to generate SO_4_^−^ and •OH, which eventually boosted large amount of ROS to cause lipid peroxidation.	[118]
(Ca, Co) CO3‐LND‐TCPP@F127‐TA	Breast cancer (4T1)	Co^2+^/Co^3+^ catalyzed the generation of O_2_^•−^ and •OH from H_2_O_2_ to consumed GSH; mitochondrial Ca^2+^ overload and SDT intensified oxidative stress.	[119]
CoOx‐loaded amorphous metal‐organic framework	Breast cancer (4T1)	Co^3+^ was reduced to Co^2+^ by GSH, thus deactivating GPX4 and enhancing the antitumor effect of ^1^O2 produced by SDT.	[120]
*Molybdenum‐based nanoparticles or nanoenzymes inducing ferroptosis*			
Tetragonal barium titanate MoS_2_ peroxidase‐like nanoenzyme	CRC (CT‐26), Breast cancer (4T1), Lung cancer (A549), HCC (HepG2)	Under USc excitation, Mo^4+^ intensively oxidized GSH and H_2_O_2_ decomposed to generate ROS in a pH‐responsive manner.	[121]
Albumin‐mediated biomimetic molybdenum sulfide nanocatalyst	Breast cancer (4T1, MCF‐7)	Mo^6+^ and Mo^4+^ respectively react with H_2_O_2_ to produce ^1^O2 and O_2_^·−^, thereby causing peroxidation of PUFAs.	[122]
*Vanadium‐based nanoparticles or nanoenzymes inducing ferroptosis*			
Vanadium‐Based Nanoplatform with the camouflage of PD‐L1inhibiting peptide	Melanoma (B16F10)	Inclusive of V^4+^ and V^5+^, when the former was oxidized, it generated •OH for lipid peroxidation; while when the latter was reduced, it consumed GSH to inactivate GPX4.	[123]
Biodegradable vanadium disulfide (VS_2_) nanosheet	CRC (CT‐26), Breast cancer (4T1), Melanoma (B16)	The V^3+^ and V^4+^ within nanoparticles could be reduced by GSH, thereby resulting in the depletion of antioxidants.	[124]
Vanadium carbide MXene nanozyme	Triple‐negative breast cancer (TNBC) (MDA‐MB‐231)	Exhibited ATPase‐like activity catalyzing the dephosphorylation of ATP to generate ADP, which led to energy exhaustion and hampered the NRF2 antioxidant signaling.	[125]

CRC, Colorectal Cancer; GBM, Glioblastoma; LUAD, Lung Adenocarcinoma; HCC, Hepatocellular Carcinoma; ALL, Acute Lymphoblastic Leukemia

### 3.4 Copper Metabolism and Ferroptosis Suppressors of Cancer

#### 3.4.1 Copper Metabolism and the system xc^−^‐GSH‐GPX4 axis

Cancer cells mainly uptake cystine through the xc^−^ system, and the latter is catalyzed by thioredoxin reductase (TxrR) within the cells and reduced to cysteine by NADPH, thereby providing the most significant reducing precursor for the synthesis of GSH.^[^ ^126^ ^]^ Cu^2+^ facilitated the nuclear translocation of HIF1A, thereby augmenting the expression of the functional subunit SLC7A11. This partially elucidated that the resistance to ferroptosis induced by ionizing radiation was mediated by the copper feedback mechanism.^[^ ^35^ ^]^ However, a copper ionophore, elesclomol, promoted the degradation of ATP7A and resulted in the retention of cellular and mitochondrial Cu^2+^, further leading to the degradation of SLC7A11 and eventually inducing ferroptosis.^[^ ^127^ ^]^ The negative modulation of SLC7A11 by high intracellular concentrations of Cu^2+^ was further verified in another studies.^[^ ^90^, ^128^ ^]^ These diverse outcomes indicate that SLC7A11 is likely to be precisely regulated by the concentration of copper ions. However, at present, the regulatory role of copper in the pathways associated with the synthesis and regeneration of GSH remains unclear.

**Figure 5:**
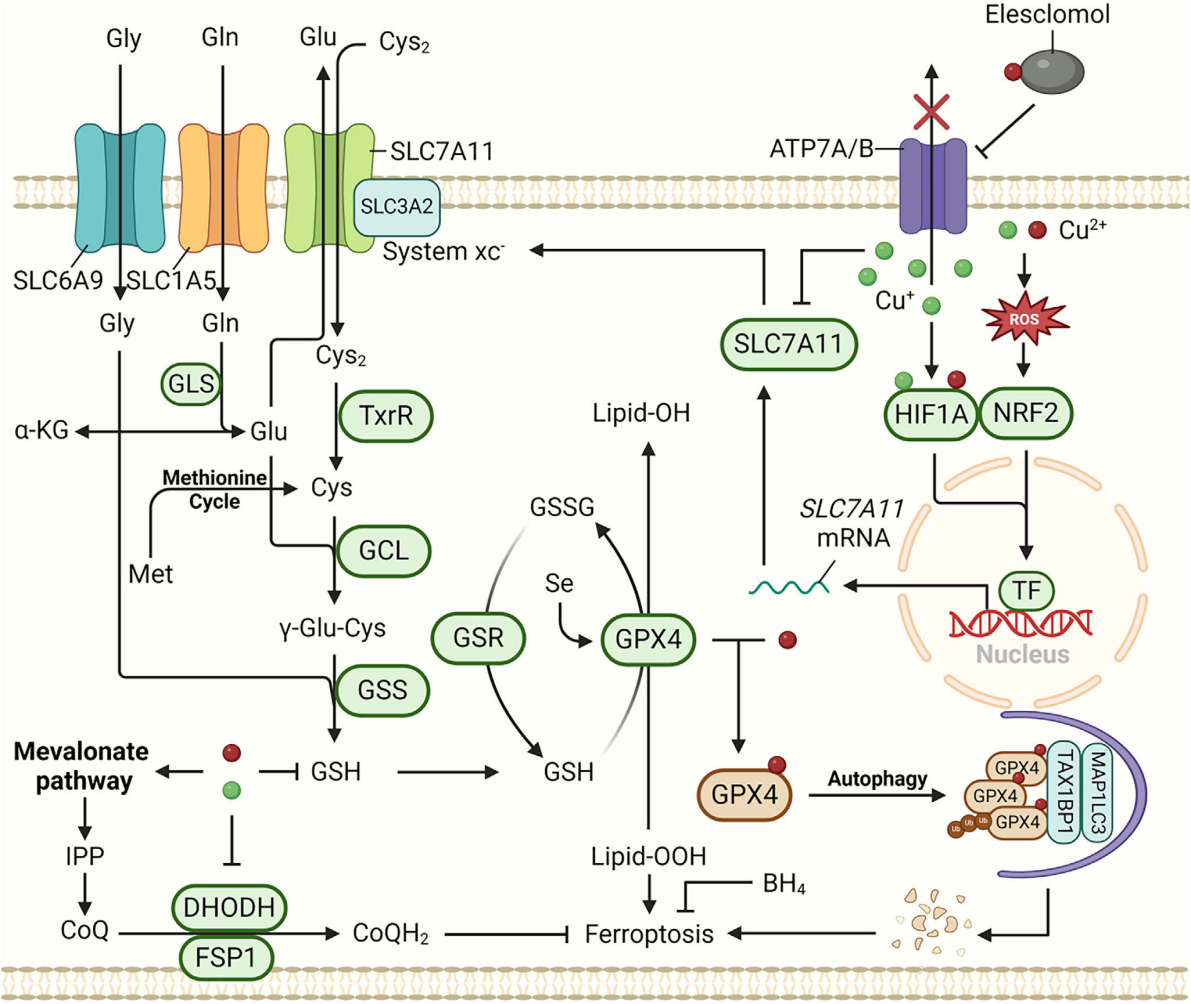
Copper metabolism and ferroptosis suppressors. Copper can directly bind and activate HIF1A, or indirectly activate NRF2 through oxidative stress to promote the expression of antioxidant genes. However, copper overload caused by copper ionophores can also facilitate the degradation of SLC7A11. The effect of Cu^2+^ binding and facilitating the autophagic degradation of GPX4 has pioneered the regulation of GPX4 by copper. Although copper can impose restrictions on GSH, the evidence of its direct influence on GSH biogenesis is still scarce. Additionally, copper can regulate the mevalonate pathway and the synthesis of CoQH_2_, but whether these processes directly affect ferroptosis is not yet clear. The green patterns in the figure represent antiferroptotic molecules.

GPX4 is one of the most significant molecules safeguarding cells against oxidative stress and currently, most metal complex‐induced ferroptosis ultimately functions through influencing GPX4.^[^ ^72^ ^]^ The regulation of GPX4 by copper is highly correlated with ferroptosis. Cu^2^⁺ could directly bind to the C107/148 cysteine residues of GPX4, leading to subsequent ubiquitination and aggregation of GPX4, which was then accepted by the autophagic receptor Tax1 binding protein 1 (TAX1BP1) and degraded in the autophagosome.^[^ ^129^ ^]^ The copper‐dependent degradation of GPX4 elicited ferroptosis in pancreatic cancer cells, a process that could be alleviated by the copper chelator tetrathiomolybdate or ferroptosis inhibitors ferrostatin‐1/liproxstatin‐1, yet not by other types of cell death inhibitors. However, the addition of ferroptosis inducers on the basis of copper salts achieved the effect of tumor eradication. In several other cancers, the downregulation of GPX4 expression was likewise observed subsequent to the introduction of exogenous copper.^[^ ^57^, ^129^, ^130^ ^]^ These results suggest that the combination of copper ionophores, copper salts, or copper nanomaterials with ferroptosis inducers (broadly speaking, antioxidant scavengers) may become a potential strategy for cancer treatment. Conversely, the inhibition of GPX4 with ferroptosis inducers erastin or RSL3 also influenced the intracellular Cu^2+^ level, which might be accomplished by modulating the expression of ceruloplasmin^[^ ^36^ ^]^ (**Figure** **5**).

#### 3.4.2 Copper Metabolism and the GPX4‐Independent Antiferroptotic Pathways

Apart from GPX4, recent studies have further revealed other antiferroptotic mechanisms, suggesting that cells have multiple radical‐trapping antioxidant systems. Metabolism‐related molecules or products assume significant importance in this process.^[^ ^131^ ^]^ FSP1, an oxidoreductase that reduces coenzyme Q (CoQ, also known as ubiquinone) to ubiquinol (CoQH_2_), has been reported to play a key role in suppressing ferroptosis together with GPX4.^[^ ^132^ ^]^ Another significant CoQ reductase is DHODH that is located in the inner mitochondrial membrane, and it is likewise capable of protecting cancer cells from ferroptosis.^[^ ^133^ ^]^ In the liver and kidney toxicity induced by copper exposure, the expression of FSP1 and DHODH declines significantly, whereas it remains unclear whether copper would exert a similar impact on cancer cells.^[^ ^134^ ^]^ However, the role of DHODH in ferroptosis resistance has been challenged. Eikan et al. discovered that the initially reported DHODH inhibitor brequinar actually operated by suppressing FSP1 at high concentrations, and the deletion of DHODH did not have such a potent sensitizing effect on RSL3. This issue calls for a meticulous investigation of DHODH functionally in cancer cells.^[^ ^133^, ^135^ ^]^ Anyway, it can be speculated that the CoQH_2_ pool is of great significance for maintaining redox homeostasis of cancer cells. Two recent studies have demonstrated that 7‐dehydrocholesterol, an intermediate of the distal cholesterol biosynthesis, functions as a direct radical trapping antioxidant both in plasma membrane and mitochondria and confers protection to cancer cells. Meanwhile, its metabolic enzyme, 7‐DHC reductase (DHCR7), exhibits a proferroptotic role. Future research ought to further investigate the influence of copper metabolism on 7‐DHC metabolism.^[^ ^19^ ^]^

Furthermore, the intermediate product of the mevalonate pathway, isopentenyl pyrophosphate (IPP), serves as a precursor for the synthesis of CoQ.^[^ ^131^ ^]^ Although it has been verified that copper could upregulate the expression of molecules in this pathway, such as 3‐hydroxy‐3‐methyl glutaryl coenzyme A (HMG‐CoA), IPP isomerase and squalene synthase, and some products like CoQ and squalene are regarded as having antilipid peroxidation effects, it remains ambiguous how copper's regulation of this pathway influences ferroptosis due to the simultaneous increase in the final product cholesterol (which is known to increase the susceptibility to ferroptosis).^[^ ^131^, ^136^ ^]^ Other than functioning as a cofactor for multiple enzymes, BH4 is also a momentous antioxidant eliminating lipid peroxyl radicals.^[^ ^3^, ^131^ ^]^ The first and rate‐limiting step of BH4 synthesis is catalyzed by GCH1 that acts as a compensatory resistance against ferroptosis when GPX4 is inhibited.^[^ ^137^ ^]^ Still, there is currently no evidence to indicate an interaction between copper metabolism and BH4 synthesis. More research is needed to uncover the relationship between them (Figure 5).

### 3.5 Copper Metabolism and Other Regulators of Ferroptosis in Cancer

#### 3.5.1 Ferroptosis‐Related Transcription Factors

NRF2 is a crucial transcription factor in response to oxidative stress through modulating the expressions of numerous antioxidant genes so as to prevent lipid peroxidation and inhibit ferroptosis.^[^ ^138^ ^]^ Although copper might not directly regulate NRF2, copper‐based therapeutics could potentially trigger the activation of NRF2.^[^ ^139^ ^]^ For instance, in hepatocellular carcinoma, renal cell carcinoma and oral squamous cell carcinoma, the negative feedback mechanisms mediated by NRF2 is capable of alleviating ferroptosis induced by DSF‐copper, thus impairing the therapeutic outcome.^[^ ^140^ ^]^ Consequently, the inhibition of NRF2 might prominently enhance the proferroptotic effect of copper‐based therapy on cancer cells. Nevertheless, disulfiram‐copper exceptionally inhibited the activation of NRF2 induced by sulfasalazine (a ferroptosis inducer inhibiting system xc^−^) and the expression of its downstream antioxidant molecules such as SLC7A11, thereby exerting a synergistic effect in lung adenocarcinoma.^[^ ^141^ ^]^ Similar inhibitory effects have also been identified in acute myeloid leukemia.^[^ ^142^ ^]^ This might be ascribed to the specificity of cancer types or the context of experimental treatment. By the way, the copper transporter ATP7A gave rise to therapeutic resistance by extruding oxaliplatin in gastric cancer, while the siRNA‐induced silencing of NRF2 is able to counterbalance this phenomenon.^[^ ^143^ ^]^

**Figure 6:**
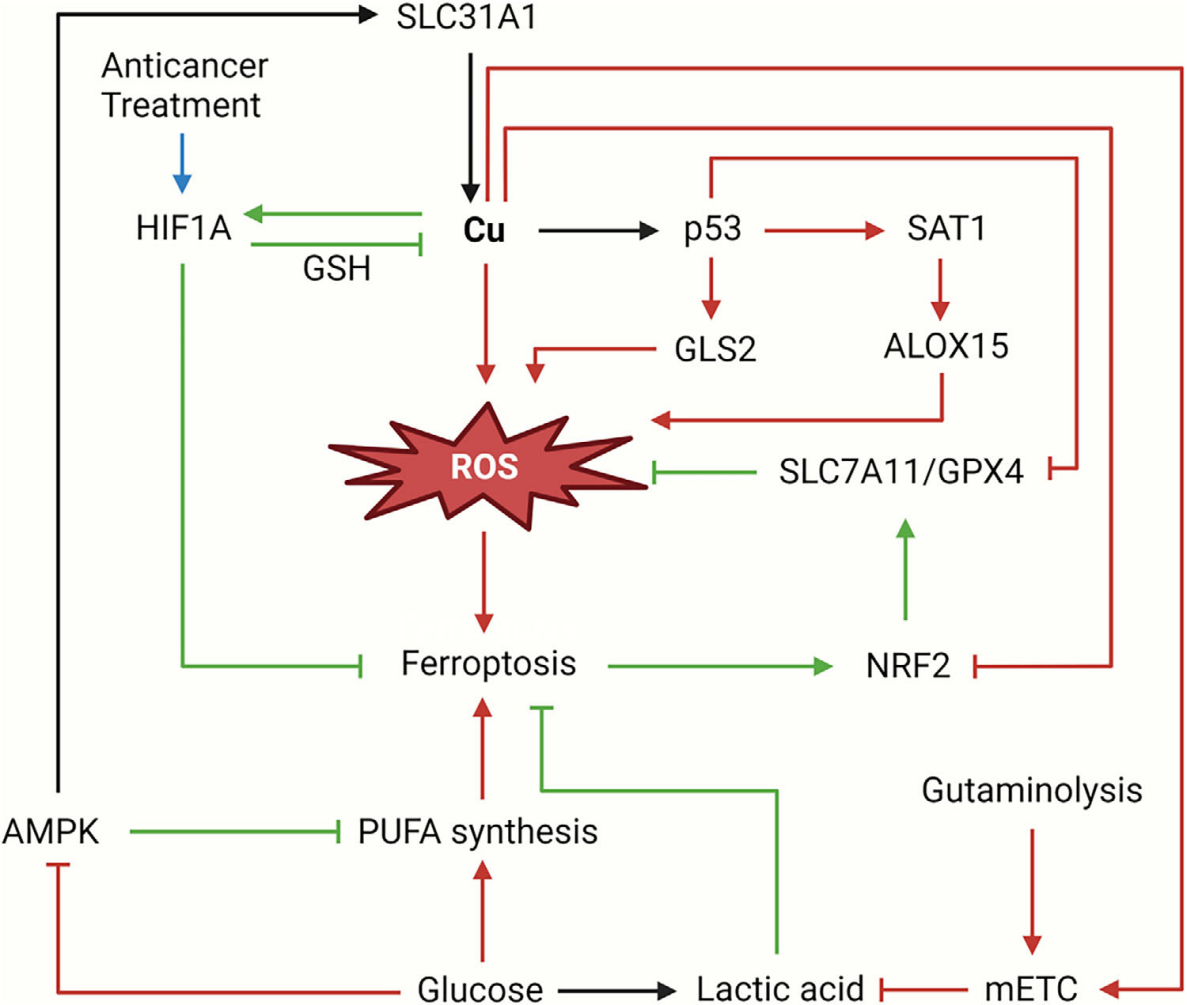
Copper metabolism and other ferroptosis regulators. In addition to NRF2 and HIF1A, another crucial tumor suppressor p53 is also a key transcription factor regulating ferroptosis. The effect of copper in facilitating ferroptosis via p53 is depicted in the figure. Metabolic pathways, particularly glucose metabolism, can markedly influence the synthesis of PUFAs. Moreover, the product of glycolysis, lactic acid, is capable of inhibiting ferroptosis. The interplay between copper and numerous ferroptosis modulators jointly constitutes a complex regulatory network. In the figure, the red line segments denote the regulatory process promoting ferroptosis, while the green ones indicate the inhibition of ferroptosis.

As for other transcription factors, HIF is regarded as being closely associated with iron homeostasis and tumor ferroptosis.^[^ ^3^, ^144^ ^]^ As previously stated, Cu^2+^ exerted an antiferroptotic effect by activating HIF1A, which was in accordance with the earlier research.^[^ ^35^, ^145^ ^]^ HIF could, conversely, regulate the concentration level of copper within the cell. Specifically, chemotherapy‐induced HIF1 activation promoted the expression of antiferroptotic genes SLC7A11 and Glutamate‐Cysteine Ligase Modifier Subunit (GCLM), thereby enhancing the synthesis of intracellular GSH to chelate copper.^[^ ^32^, ^146^ ^]^ This implies a regulatory loop between HIF signaling and copper. P53, the most important tumor suppressor, exerts a role in either promoting or inhibiting ferroptosis under diverse circumstances.^[^ ^3b^ ^]^ DSF‐Cu induced p53‐mediated ferroptosis in nasopharyngeal carcinoma cells.^[^ ^147^ ^]^ In p53‐deficient nonsmall cell lung cancer (NSCLC) cells, high expression of WEE1 G2 Checkpoint Kinase enhanced the sensitivity of adavosertib and, when combined with DSF‐Cu, inhibited SLC7A11 to cause synergistic lethality.^[^ ^128^ ^]^ Two other transcription factors associated with ferroptosis, BRCA1 Associated Protein 1 (BAP1) and Yes1 Associated Transcriptional Regulator (YAP),^[^ ^3b^ ^]^ their interactions with copper remain unclear (**Figure** **6**).

#### 3.5.2 Noncoding RNA

In recent years, multiple noncoding RNAs have been found to be involved in the biological process of ferroptosis.^[^ ^148^ ^]^ Recently, a novel lnc‐RNA LINC02936 inhibited ferroptosis of endometrial cancer cells via the SIX Homeobox 1/ceruloplasmin axis, initially indicating the association among copper metabolism‐related proteins, noncoding RNAs and ferroptosis in cancer.^[^ ^37a^ ^]^ Regarding miRNA, there have been research on its modulatory role in copper‐mediated ferroptosis of hepatocytes, and such a role may also occur in cancer.^[^ ^134c^ ^]^

#### 3.5.3 Other Metabolic Pathways

In addition to lipid metabolism, both glucose metabolism and amino acid metabolism have been demonstrated to interact with the ferroptosis process.^[^ ^131^, ^149^ ^]^ Glucose starvation, an energy stress that deprived the main source of ATP, could significantly inhibit cell ferroptosis.^[^ ^150^ ^]^ The reason lay in that the activation of the important glucose sensor AMPK, suppressed lipogenesis, which diminished the generation of ROS within the cells.^[^ ^151^ ^]^ Interestingly, the activation of AMPK triggered by glucose starvation led to an upregulation of CTR1 expression, thus establishing a connection with copper metabolism.^[^ ^152^ ^]^ AMPK also mediated the stability of CTR1 through phosphorylation to augment copper uptake.^[^ ^153^ ^]^ Copper levels can also regulate the activity of AMPK. Studies have confirmed that copper depletion simulated by the copper chelator tetrathiomolybdate promoted the phosphorylation of AMPK and its activation.^[^ ^154^ ^]^ These results imply the potential interaction of copper and AMPK in cancer ferroptosis. It has been widely reported that the glycolytic metabolite lactic acid confers resistance to ferroptosis in tumor cells.^[^ ^155^ ^]^ Given that copper acts as an essential cofactor for multiple mitochondrial respiratory chain enzymes, it is not hard to infer that their deficiency will impact mitochondrial function and metabolic capacity, thereby resulting in an augmented production of lactic acid and influencing ferroptosis.^[^ ^7^, ^156^ ^]^ Glutaminolysis ultimately gives rise to α‐ketoglutarate (α‐KG), which fuels the TCA cycle and regulates ferroptosis.^[^ ^157^ ^]^ However, whether copper exerts an influence on this process remains undefined (Figure 6).

### 3.6 The Crosstalk between Cuproptosis and Ferroptosis

#### 3.6.1 The Intersectional Hub between Cuproptosis and Ferroptosis

**Figure 7:**
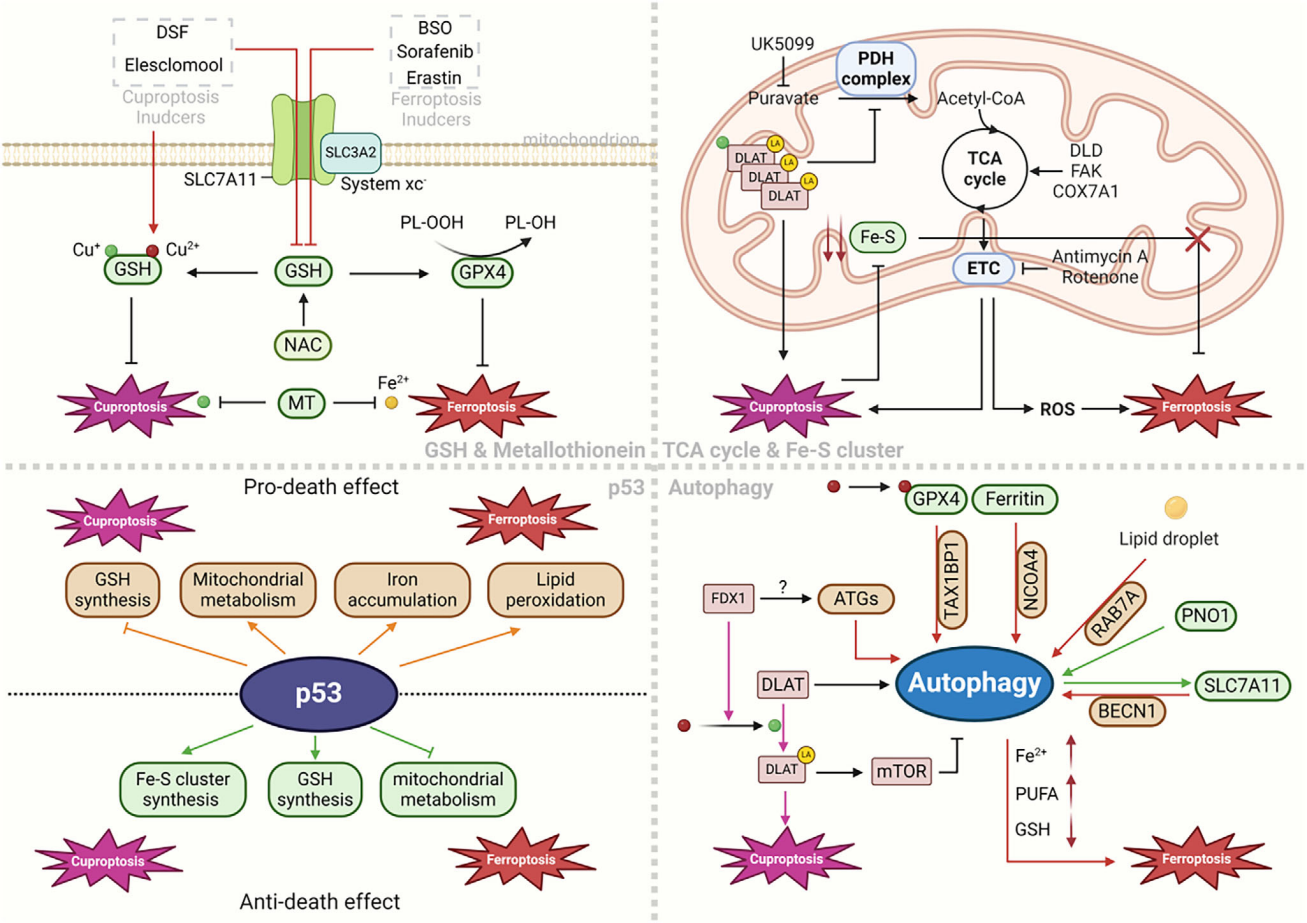
Crosstalk between cuproptosis and ferroptosis. The crosstalk between cuproptosis and ferroptosis is mainly achieved via four crucial hubs: 1) GSH and metallothionein (MT): In the process of cuproptosis, GSH mainly performs the function of sequestering toxic Cu^+^; while in ferroptosis, GSH assists GPX4 in detoxifying lipid peroxides. Cuproptosis inducers and ferroptosis inducers can realize co‐targeting via GSH. MT, on the other hand, sequesters Cu^+^/Cu^2+^ and Fe^2+^ respectively. 2) Mitochondrial TCA cycle and Fe‐S clusters: The TCA cycle and respiratory chain of mitochondria both fuel the cuproptosis and ferroptosis. The lipoylation of mitochondrial proteins during cuproptosis will impact the function of mitochondria. Moreover, the reduction of iron‐sulfur clusters triggered by cuproptosis might facilitate the process of ferroptosis. 3) p53: The most important tumor suppressor p53 exerts a dual effect on both cuproptosis and ferroptosis, mainly through the regulation of the components of the two. 4) Autophagy: Several mediators involve the autophagy of related components so as to promote ferroptosis. In cancer, the interplay between cuproptosis and autophagy is not yet very well‐defined, but there is evidence indicating an association between the two. The brown patterns in the figure represent proferroptotic molecules, while the green patterns represent antiferroptotic molecules.

As a cell death mode centered on metal elements, the related mechanisms of cuproptosis have been presented in other outstanding reviews.^[^ ^7^, ^24^ ^]^ Distinct from the oxidative stress caused by ferroptosis, the occurrence of cuproptosis is ultimately attributed to the aggregation of lipoylated proteins resulting in mitochondrial proteotoxic stress. However, in view of the similar redox properties of copper and iron, there exists some common convergence hubs between cuproptosis and ferroptosis (**Figure** **7**).

##### GSH and Metallothionein

During ferroptosis, GSH primarily acts as a kind of RTA, sustaining the activity of GPX4 for the elimination of peroxidative free radicals; whereas in cuproptosis, GSH serves as the principal copper‐sequestering substance, restricting the activity of cuprous ions and thereby reducing their combination with lipoylated Dihydrolipoamide S‐acetyltransferase (DLAT, a component of mitochondrion pyruvate dehydrogenase (PDH) complex).^[^ ^7^, ^24^ ^]^ At the initial discovery of cuproptosis, the GSH synthesis inhibitor buthionine sulfoximine (BSO) rendered cells more susceptible to copper ionophores and could induce cuproptosis independently.^[^ ^158^ ^]^ This implies, despite differing mechanisms, GSH restricts key processes in both cuproptosis and ferroptosis. For instance, in the simultaneous induction of cuproptosis and ferroptosis by elesclomol‐Cu and erastin, supplementation of GSH or its precursor N‐acetylcysteine (NAC) concurrently alleviated the severity of both.^[^ ^159^ ^]^ Restoring the expression of xc^−^ system or knocking out *Ferredoxin 1* (*FDX1*, a key gene for cuproptosis) reversed the inhibitory effects of erastin or elesclomol‐Cu on myelodysplastic syndrome (MDS) cells alone, but failed to rescue the effects of combined treatment, further proving that GSH is the intersection of the network.^[^ ^159^ ^]^ In HCC, the ferroptosis inducers sorafenib or erastin could also trigger cuproptosis characterized by upregulated lipoylated DLAT, partially through hindering the uptake of cystine by the xc^−^ system to reduce intracellular GSH abundance.^[^ ^160^ ^]^ Recently, GSH was found to contribute to the resistance of cells to cuproptosis, while GPX4 inhibitor RSL3 and simvastatin, could reduce intracellular GSH levels to restore sensitivity.^[^ ^161^ ^]^ Similarly, as previously mentioned, the cuproptosis inducers could also target the GSH synthesis system or boost oxidative stress to facilitate ferroptosis.^[^ ^57^, ^127^, ^140^, ^162^ ^]^ To sum up, there are associations between cuproptosis and ferroptosis via GSH. Targeting GSH may enhance the efficacy of one of the mechanisms or simultaneously induce both types of cell death, thereby offering a promising strategy for cancer treatment.

Metallothionein, another substance capable of sequestering toxic metals and neutralizing ROS, plays a significant role in the defense of metallic oxidative stress. Current evidence indicates that MTs and related regulatory factors are involved in the resistance of cancer cells to ferroptosis.^[^ ^163^ ^]^ Although direct evidence for MT regulating cuproptosis has not yet been obtained, it can be speculated that copper‐bound MT might function through a similar manner.^[^ ^7a^ ^]^ Moreover, the activity of MT per se is modulated by GSH, and both are jointly governed by the NRF2 antioxidant signaling, thus linking cuproptosis with ferroptosis^[^ ^7^, ^163^ ^]^ (Figure 7).

##### TCA Cycle and Iron–Sulfur Cluster in Mitochondria

Mitochondria are the most essential organelles for energy metabolism in mammals. The extensive transfer of electrons between oxygen atoms, along with the multiple enzymes associated with oxidative phosphorylation, collectively establish them as the primary source of ROS.^[^ ^164^ ^]^ Consequently, mitochondria constitute the principal site driving ferroptosis.^[^ ^165^ ^]^ The production of ROS not only leads to damage to mitochondria characterized by the swelling of the mitochondrial shape, disappearance of the membrane potential, increased permeability of the membrane and a reduction of cristae, but also affects the redox status of other compartments of the cell.^[^ ^12^, ^165^ ^]^ Copper plays a leading role in the TCA cycle and the electron transfer chain (ETC).^[^ ^166^ ^]^ Hence, cuproptosis also takes place in the mitochondria. Unlike ferroptosis, cuproptosis is characterized by the accumulation of toxic Cu⁺ within cellular mitochondria, coupled with the exposure of hydrophilic groups induced by its binding to DLAT in the PDH complex, ultimately leading to protein aggregation.^[^ ^7^, ^156^ ^]^ Although the mitochondrial morphological alterations during cuproptosis are distinct from those in ferroptosis, usually shrunken,^[^ ^166^ ^]^ we can still capture the interplay between the two from the TCA cycle and ISCs.

In the initial research, both mitochondrial pyruvate carrier inhibitors and ETC inhibitors were capable of alleviating the cuproptosis induced by copper ionophores. Simultaneously, lung cancer cell line NCIH2030 relying on mitochondrial respiration demonstrated higher sensitivity to elesclomol‐Cu compared to those dependent on glycolysis.^[^ ^158a^ ^]^ Furthermore, the HCC cells HepG2 and Hep3B lacking AT‐Rich Interaction Domain 1A (ARID1A) demonstrated a pronounced dependence on mitochondrial respiration and oxidative phosphorylation, along with a significant sensitivity to copper treatment.^[^ ^167^ ^]^ Similar results have also been reported in gastric cancer cells with low expression of Integrin β1 (ITGB1).^[^ ^168^ ^]^ These results indicate that cuproptosis is TCA cycle and ETC‐dependent. Concerning ferroptosis, mitochondria likewise exert a regulatory role in ferroptosis induced by cysteine deprivation. Inhibiting the TCA cycle or the ETC could both mitigate ferroptosis.^[^ ^169^ ^]^ Additionally, the fact that glutaminolysis or other regulators such as dihydrolipoamide dehydrogenase (DLD), focal adhesion kinase (FAK) and COX7A1 fuel the TCA cycle to potentiate ferroptosis in different cancers further substantiates this perspective.^[^ ^157^, ^170^ ^]^ Like ferroptosis, cuproptosis can also influence the TCA cycle conversely. Apart from the direct binding of Cu^+^ to DLAT, other enzymes related to the lipoic acid pathway or the PDH complex during cuproptosis also exhibit an increase in lipoylation levels, jointly influencing the carbon flux entering the TCA cycle.^[^ ^158^, ^171^ ^]^ Since DLAT is indispensable for catalyzing the production of acetyl‐CoA in the pyruvate dehydrogenation reaction, its lipoylation might compromise this function and thereby disrupt the TCA cycle. Nevertheless, due to the intricacy of the regulatory network governing ferroptosis, the influence of this process on ferroptosis and its degree remain ambiguous.

ISCs, as a vital constituent for maintaining mitochondrial iron homeostasis as well as a type of antioxidant, exert a key role in safeguarding cells against ferroptosis.^[^ ^3a^ ^]^ Owing to the elevated level of ROS, tumor cells typically exhibit significant dependence on ISCs, thus positioning them as potential targets for cancer therapy.^[^ ^172^ ^]^ The functional mechanisms of various proteins related to ISCs in defending against ferroptosis have been concretely investigated.^[^ ^172b^ ^]^ One characteristic of cuproptosis is the destabilization of ISCs.^[^ ^158a^ ^]^ Therefore, the prevailing opinion holds that the degradation of ISCs (this might be coupled with ferroptosis) is a consequence of cuproptosis, rather than the initiating factor. However, studies have also suggested that ISCs furnish the requisite sulfur atoms for the catalysis of lipoic acid synthetase (LIAS). Consequently, the downregulation of ISCs may suppress the synthesis of lipoic acid and thereby impede lipoylation.^[^ ^7a^ ^]^ This indicates that ISCs might be a crucial factor in the negative feedback loop of cuproptosis and discloses a mechanism distinct from that in ferroptosis (Figure 7).

##### P53

P53 is one of the most pivotal transcription factors regulating cell death, and modulation of cuproptosis and ferroptosis by it has been extensively investigated.^[^ ^173^ ^]^ Hence, an interaction between cuproptosis and ferroptosis might occur via p53. However, these associations are frequently indirect, being reflected in the regulation by p53 on such “core effectors” of cuproptosis and ferroptosis such as the synthesis of GSH, the synthesis of ISCs, and mitochondrial metabolism. As the relevant details have been covered in other reviews,^[^ ^173^ ^]^ they will not be elaborated upon herein and can be referred to the figure (Figure 7).

##### Autophagy

Autophagy is a process by which cells engulf their own proteins or organelles to meet certain metabolic requirements and update cellular components, which serves as a critical regulator of cell death.^[^ ^174^ ^]^ Recent studies have shown that ferroptosis is modulated by autophagy‐related proteins and is autophagy‐dependent in certain circumstances.^[^ ^49^, ^175^ ^]^ For instance, processes such as NCOA4‐mediated ferritinophagy, RAB7A‐mediated lipophagy, and Beclin1 (BECN1)‐mediated inhibition of system xc^−^ can all facilitate ferroptosis.^[^ ^49^, ^175^ ^]^ Specifically, AMPK‐mediated phosphorylation of BECN1 at S93/96 promoted the formation of the BECN1‐SLC7A11 complex which pulled SLC7A11 into the autophagosome for degradation.^[^ ^176^ ^]^ This process undermined the biogenesis of GSH, thereby resulting in oxidative stress. It can be hypothesized that the activation of BECN1 might decrease the sequestration of Cu^+^ by GSH and triggered cuproptosis. Nevertheless, there exist instances where autophagy inhibits ferroptosis. In HCC, the high expression of Partner of NOB1 Homolog (PNO1) activated autophagy, but contrarily promoted the expression of SLC7A11 and the synthesis of GSH, indicating the complexity of autophagy regulatory mechanisms.^[^ ^177^ ^]^

As mentioned earlier, copper itself has a regulatory role in the autophagy process, which may affect ferroptosis directly or indirectly.^[^ ^50^, ^51^, ^52^, ^53^, ^129^ ^]^ Although the concept of cuproptosis has been proposed for a short time, existing studies have indicated the association between it and autophagy. The key protein of cuproptosis, DLAT, was discovered to potentially promote tumorigenesis of diverse cancers by stimulating autophagy, thereby endowing it with the function of an oncogene.^[^ ^178^ ^]^ However, in the cuproptosis of prostate cancer induced by elesclomol‐Cu, the lipoylation of DLAT inhibited autophagy by activating mTOR, which sensitized cells to docetaxel.^[^ ^179^ ^]^ This indicates that there might be differences in the regulation of autophagy by DLAT in the normal state and the cuproptosis state. A recent database‐based analysis revealed that the expression of another key gene FDX1 for cuproptosis is positively correlated with that of autophagy‐related genes ATG5, ATG12 and BECN1.^[^ ^180^ ^]^ However, this research failed to disclose the causal relationship between them. At present, it remains unclear whether autophagy influences cuproptosis, and further studies will contribute to deepening the understanding of the mechanisms for enhancing cuproptosis (Figure 7).

#### 3.6.2 Cotargeting Ferroptosis and Cuproptosis for Cancer Therapy

In recent years, cuproptosis and ferroptosis have drawn extensive attention and yielded remarkable achievements, among which some have been gradually applied in clinical practice.^[^ ^3^, ^181^ ^]^ Specifically, the metabolic vulnerabilities of cancer cells can be exploited to induce ferroptosis or cuproptosis, or be combined with other anticancer therapeutic approaches. Owing to the interaction between cuproptosis and ferroptosis at the aforementioned hubs, co‐targeting two pathways might offer a prospect for eradicating refractory cancer (**Table** **2**).

**Table 2:** Cotargeting ferroptosis and cuproptosis for cancer treatment.

Strategy	Cancer Type (Cell Line)	Antitumor Effect and Related Mechanisms	Refs.
Chlorin e6 @Cu Nanoparticle	GBM (U87MG)	The released Cu^2+^ from nanoparticles triggered ferroptosis and cuproptosis respectively via GSH depletion and DLAT aggregation; sonodynamic effect induced by chlorin e6 further boosted the generation of ROS.	[75]
RNP@Cu2O@SPF	CRC (HT29, SW480)	CRISPR‐Cas9‐sgATP7A facilitated the intracellular accumulation of Cu^+^; the liberated Cu^+^ promoted the lipoylation of DLAT; the Fenton reaction induced by Cu^+^ generated hydroxyl radicals to initiate lipid peroxidation.	[85]
MitCuOHA nanozyme	Cervical cancer (Hela), HCC (HepG2), Breast cancer (4T1, MCF‐7)	Cu^2+^ exhaused cysteine and GSH through the generation of H_2_O_2_ to induce ferroptosis and cuproptosis	[86]
Erastin/sorafenib+Elesclomol‐Cu	HCC (MHCC‐97H, QBC939)	Erastin/sorafenib reduced the degradation of FDX1 protein mediated by mitochondrial proteases, thereby elevating the lipoylation of DLAT; inhibited the function of system xc^−^ in GSH synthesis, consequently diminishing the sequestration of exogenous copper introduced by elesclomol.	[160]
Erastin/sulfasalazine+DSF‐Cu ATF4 knockdown+DSF‐Cu	HCC (SMMC‐7721, Huh7)	Erastin/sulfasalazine suppressed the compensatory increment of xCT resulting from DSF‐Cu treatment; ATF4 knockdown reversed the inhibitory effect of endoplasmic reticulum (ER) stress on ferroptosis.	[162]
CuP/Er nanoscale coordination polymer (CuP/Er NCP)	Breast cancer (4T1)	The erastin depleted GSH by inhibiting system xc^−^ and, opened the voltage‐dependent anion channels (VDACs) on the outer mitochondrial membrane leading to a change in membrane potential and inhibition of glycolysis (the Warburg effect), thus increasing the production of mitochondrial ROS; copper itself promoted the lipoylation of DLAT. It also synergized with PD‐L1 antibodies to repolarize macrophages from M2 to M1 phenotype to promote antigen presentation and antitumor immunity.	[182]
Cu_2_O@Mn_3_Cu_3_O_8_ (CMCO) nanozyme	CRC (CT26)	The synergy of Cu^+^ and Mn^2+^ initiated the Fenton‐like reaction to trigger ferroptosis; Cu^+^ promoted the aggregation of DLAT to initiate cuproptosis.	[183]
*E. coli*@Cu_2_O microbial nanohybrid	CRC (MC38)	*E. coli* targeted colon tumors, where Cu_2_O consumed endogenous H_2_S, and the generated Cu^+^ inactivated GPX4 and promoted the aggregation of DLAT.	[184]
Cellular Trojan Horse Lip@Fe‐Cu Metal‐organic framework	Breast cancer (4T1)	Live neutrophils stabilized thermosensitive liposomal bimetallic framework, which evaded immune surveillance and tended toward tumors where NPs were taken up by cells and released Fe^3+^/Cu^2+^ to cause mitochondrial damage.	[185]
Sodium alginate coencapsulated Cu‐Fe_3_O_4_ nanozyme and artemisinin	Osteosarcoma (143b)	Released Fe^2+^/Cu^2+^ reacted with artemisinin to generated carbon radicals (•C) that amplified oxidative stress, which induced apoptosis/ferroptosis/cuproptosis.	[186]
DSF@HMCIS‐PEG‐FA	Breast cancer (4T1) Gastric cancer (HGC‐27)	Released DSF, H_2_S, Cu^2+^, and Fe^2+^ in the acid tumor microenvironment and DSF amplified Fenton/Fenton‐like reactions to kill tumor cells.	[187]
CuMoO4 nanodot	Breast cancer (4T1, MCF‐7)	The reduction of Cu^2+^ and Mo^6+^ jointly consumed GSH, thereby inducing copperptosis and ferroptosis.	[188]

CRC, Colorectal Cancer; GBM, Glioblastoma; HCC, Hepatocellular Carcinoma

## 4 Other Metal‐related Mechanisms of Ferroptosis in Cancer

### 4.1 Zinc‐Mediated Ferroptosis in Cancer

As the transition metal whose abundance is second only to that of iron, zinc is crucial for the redox balance of cells.^[^ ^189^ ^]^ Both deficiency and excess of zinc can induce oxidative stress so it is defined as a “pro‐antioxidant”.^[^ ^189^ ^]^ Zinc also serves as an important cofactor for SOD together with copper. Owing to the greater ease of “adding” zinc, multiple zinc‐based nanomaterials have been exploited for inducing ferroptosis.^[^ ^108^, ^109^, ^110^, ^111^, ^112^ ^]^ Since Zn^2+^ is the sole ionic form within cells, the depletion of GSH by Zn‐based nanomaterials is presumably not accomplished through direct redox processes.^[^ ^190^ ^]^ However, Zn^2^⁺ can form a coordination bond with the sulfhydryl group of cysteine residues, thereby modulating redox state of corresponding molecules.^[^ ^191^ ^]^ This may partially explain the mechanism by which zinc induces oxidative stress. Indeed, excessive ZnCl_2_ induced the death of A549 lung cancer cells not by means of apoptosis or necrosis, but rather through ferroptosis accompanied by lipid peroxidation.^[^ ^192^ ^]^ This might be attributed to the substitution of iron in the mitochondrial ETC by zinc, thereby resulting in an increase in the labile iron pool.^[^ ^192^ ^]^ Recently, a novel mechanism by which zinc independently induces ferroptosis during iron chelation has been clarified.^[^ ^8b^ ^]^ Mechanistically, knockout of ZIP7 (zinc transporter protein 7, encoded by *SLC39A7*) led to accumulation of zinc in the endoplasmic reticulum (ER) and ER stress, which subsequently induced the expression of Homocysteine Inducible ER Protein with Ubiquitin Like Domain 1 (HERPUD1) and Activating Transcription Factor 3 (ATF3). This protected renal cancer cells and breast cancer cells from ferroptosis, whereas Zn supplementation will abolish this protective effect.^[^ ^8b^ ^]^ Furthermore, zinc deficiency was likely to mediate ferroptosis resistance in esophageal squamous cell carcinoma through promoting glycolysis and lactate generation, subsequently inhibiting AMPK and upregulating the Sterol Regulatory Element‐Binding Protein 1 (SREBP1)/ Stearoyl‐CoA Desaturase 1 (SCD1) axis.^[^ ^193^ ^]^ This further enriches the role of zinc in ferroptosis. However, although previous studies have indicated that zinc can alleviate ferroptosis in other cells, its role in cancer cells remains undefined.^[^ ^4^, ^189^ ^]^

**Figure 8:**
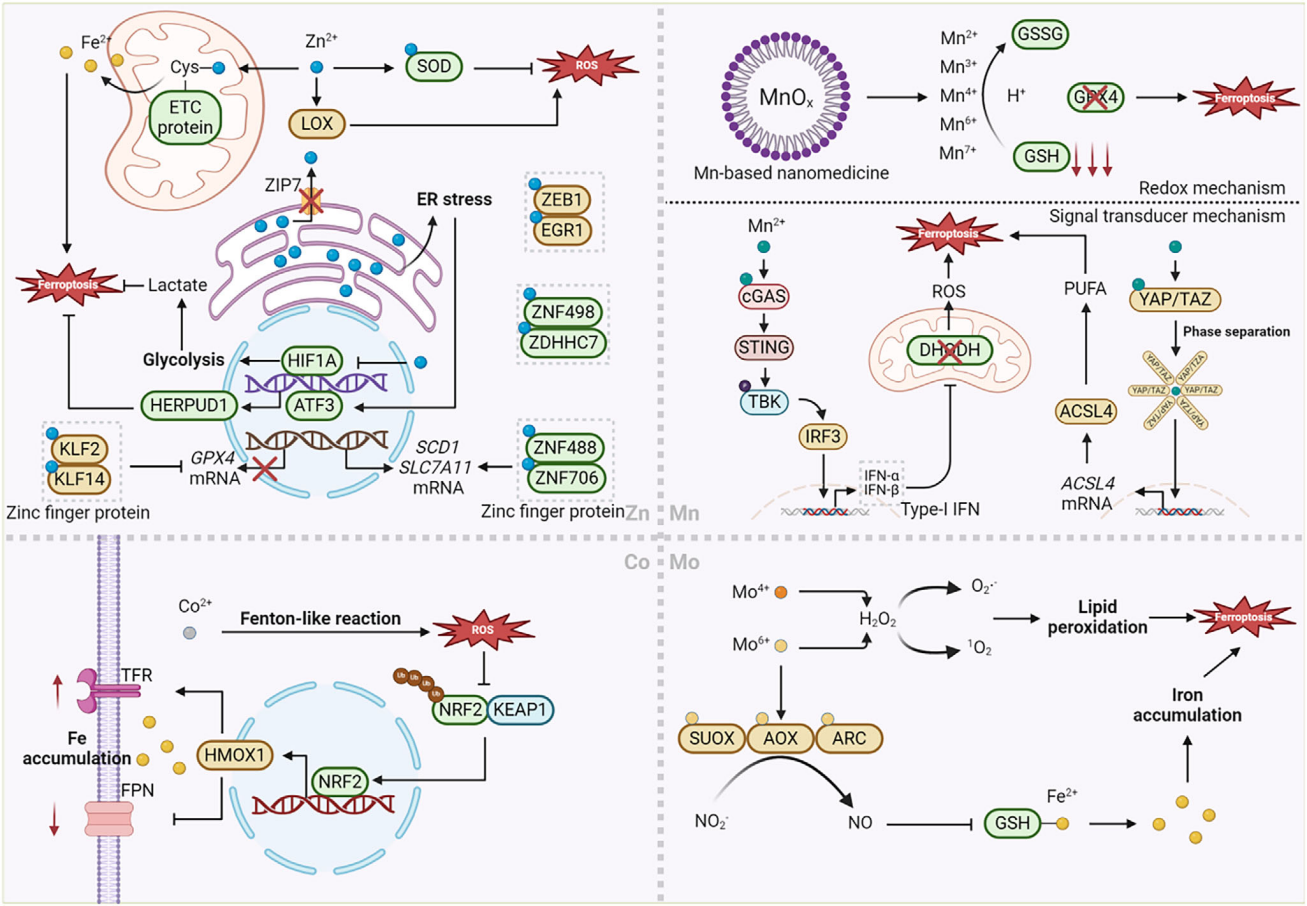
Other metal‐mediated ferroptosis in cancer. 1) Zinc: The mechanism of zinc‐mediated ferroptosis mainly lies in the coordination substitution with cysteine residues and the alterations in the activity of transcription factors induced by ER stress. Different zinc finger proteins are also capable of promoting or inhibiting ferroptosis through the regulation of gene transcription. 2) Manganese: Traditionally, manganese consumes GSH through direct oxidative reactions. Mn^2+^ is also able to promote ferroptosis by activating interferon responses and the phase separation of transcription factors. 3) Cobalt: Cobalt‐induced oxidative stress activates the NRF2/HMOX1 axis to increase intracellular iron pool abundance and induce ferroptosis. (4) Molybdenum: Mo^4+^ and Mo^6+^ react with H_2_O_2_ to produce ROS. MoO_4_^2−^ is also capable of facilitating the generation of intracellular NO in order to restrict the sequestration of Fe^2+^ by GSH. The brown patterns in the figure represent proferroptotic molecules, while the green patterns represent antiferroptotic molecule.

The zinc finger motif is a protein structural unit that is widely present in eukaryotic cells. It is formed through coordination between zinc and cysteine residues and possesses numerous biological functions (usually DNA‐binding proteins).^[^ ^194^ ^]^ The influence of zinc on ferroptosis is also indirectly manifested in some zinc finger proteins identified in recent years. For instance, Krueppel‐Like Factor 14 (KLF14) facilitated ferroptosis in cervical cancer by suppressing the transcription of the *GPX4* gene. However, deletion of its zinc finger motif deprived it of the ability to bind to the *GPX4* promoter.^[^ ^195^ ^]^ KLF2 low expression was associated with poor prognosis of colorectal cancer (CRC) patients. The overexpression of KLF2 inhibited the proliferation and metastasis of SW480 cells by suppressing the PI3K/AKT/GPX4 axis.^[^ ^196^ ^]^ Another zinc finger protein ZNF488, however, promoted the proliferation of pancreatic cancer cells by enhancing the transcription activity of *SCD1* gene to inhibit ferroptosis.^[^ ^197^ ^]^ Similarly, ZNF706 enhanced ferroptosis resistance by promoting the transcription of the *SLC7A11* in HCC.^[^ ^198^ ^]^ Although it remains unclear whether the interactions between zinc finger‐containing proteins and ferroptosis are all associated with Zn^2+^, their proferroptotic (Zinc Finger E‐Box Binding Homeobox 1 (ZEB1), Early Growth Response 1 (EGR1))^[^ ^199^ ^]^ or antiferroptotic (ZNF498, Zinc Finger DHHC‐Type Palmitoyltransferase 7 (ZDHHC7))^[^ ^200^ ^]^ roles have been extensively reported. These results reflect the distinct functions and cancer specificity of different zinc finger‐containing proteins. Furthermore, zinc might modulate ferroptosis through influencing the metabolism of iron and the activity of oxidoreductase enzymes^[^ ^189^ ^]^ (**Figure** **8**).

### 4.2 Manganese‐Mediated Ferroptosis in Cancer

Manganese oxide nanomaterials have been widely developed for inducing ferroptosis in cancer and enhancing the efficacy of other treatments such as PDT, CDT, SDT, and PTT (Table 1).^[^ ^201^ ^]^ A prevalent phenomenon is that these nanoplatforms result in the inactivation of GPX4 and the depletion of GSH, and eventually the generation of a large quantity of ROS. A prevailing explanation is that manganese possesses multiple valence states, enabling it to form diverse manganese oxides and display distinct chemical properties in diverse environments.^[^ ^201^ ^]^ This is the reason why manganese oxide nanomaterials mainly consist of Mn^3+^ or Mn^4+^, exploiting their strong oxidizing properties under acidic conditions to react with the antioxidant GSH. Recently, other exact mechanisms of manganese have also been found to be involved in cancer ferroptosis. In a variety of cancer cells, Mn^2+^ bound to cyclic GMP‐AMP Synthase (cGAS) and activated the Stimulator of Interferon Response cGAMP Interactor 1 (STING)/TANK Binding Kinase 1 (TBK1)/ Interferon Regulatory Factor 3 (IRF3) axis.^[^ ^9a^ ^]^ The type‐I interferon (IFN) thereby released significantly suppressed the expression of DHODH, resulting in the breakdown of mitochondrial ferroptosis defense and exerting antitumor effects.^[^ ^9a^ ^]^ The conclusion that Mn^2+^ activated cGAS‐STING pathway has been further verified in the application of manganese‐containing nanoplatforms.^[^ ^202^ ^]^ Specifically, Mn^2+^ and other metal ions synergistically activated the IFN signaling pathway of dendritic cells and facilitated the activation of CD8⁺ T cells.^[^ ^202a,b^ ^]^ Meanwhile, they could induce ferroptosis in cancer cells and enhance the antitumor immunity. Mn^2+^ was also capable of binding to the transcription factors YAP/TAZ, expediting their phase separation and subsequent nuclear translocation. This process upregulated the expression of *ACSL4* and promoted the synthesis of PUFAs, which derived ferroptosis in oral squamous cell carcinoma.^[^ ^9b^ ^]^ This indicates that manganese can act as a signal transducer, rather than solely triggering oxidative stress via redox reactions. Overall, the current studies concerning manganese all demonstrate its promoting effect during the process of ferroptosis, regardless of the specific mechanisms.^[^ ^203^ ^]^ Consequently, further exploration of the mechanisms through which manganese triggers ferroptosis and the development of novel manganese‐based bioactive materials are conducive to more effective targeting of ferroptosis (Figure 8).

### 4.3 Cobalt‐Mediated Ferroptosis in Cancer

Similar to copper and manganese, cobalt is frequently employed in nanomaterials for inducing redox reactions because of its electronic arrangement characteristics, diversity of valence states and extensive metal‐ligand bonding properties (Table 1).^[^ ^116^, ^117^, ^204^ ^]^ It also markedly potentiates the proferroptotic function of Fe or Zn‐based nanomaterials.^[^ ^205^ ^]^ Although cobalt is a cofactor of vitamin B_12_, excessive cobalt can initiate the Fenton‐like reaction to generate ROS and cause detrimental effects.^[^ ^206^ ^]^ Cobalt‐60 has been extensively employed in clinical radiotherapy, and cobalt‐based nanomaterials are capable of enhancing the sensitivity of radiotherapy through inducing ferroptosis.^[^ ^116^ ^]^ In this research, Co^2+^ could upregulate HMOX1 and then resulted in the increase of TFR and the decrease of FPN, and ultimately giving rise to iron accumulation.^[^ ^116^ ^]^ However, it remains uncertain whether cobalt, similar to manganese, serves as a signal transducer to modulate other components of ferroptosis and deserves further exploration (Figure 8).

### 4.4 Molybdenum‐Mediated Ferroptosis in Cancer

Molybdenum is another essential metallic element with various valence states (+3, +4, +5, +6) for the human body and acts as a cofactor for several oxidoreductases such as sulfite oxidase (SUOX), aldehyde oxidase (AOX), and mitochondrial amidoxime‐reducing component (mARC).^[^ ^207^ ^]^ In ovarian cancer cells treated with sodium molybdate, an increase in labile iron pools and depletion of GSH were observed, which was mediated by MoO_4_^2−^ activating these enzymes to catalyze the production of NO_2_^−^ to generate NO^[^ ^208^ ^]^. Furthermore, a biomimetic molybdenum sulfide nanocatalyst has been developed recently, which is capable of catalyzing the formation of singlet oxygen (^1^O_2_) and superoxide anions (O_2_^•−^) from H_2_O_2_, and subsequently activating ferroptosis. This offers a reference for the novel application of metals in cancer therapy^[^ ^122^ ^]^ (Figure 8).

## 5 Conclusions and Future Perspectives

Trace metal elements are not merely essential for the human body but also exert significant influences in various biological processes such as the genesis and progression of cancer.^[^ ^1^ ^]^ Metal elements like iron, copper, and zinc, through their participation in biological processes including cell signal transduction, gene expression regulation, and free radical scavenging, have an impact on the survival and mortality of cancer cells. Ferroptosis, as a novel form of regulated cell death (RCD), which is highly context‐dependent in cancer therapy, has received intense attention over the past decade and achieved remarkable research outcomes.^[^ ^3b–d^ ^]^ Interestingly, an increasing number of mechanisms involved in the regulation of the ferroptosis process (namely, iron accumulation, lipid peroxidation, and ferroptosis suppressors) have been identified, including those seemingly “negligible” nonferrous trace metal elements, which are also the objects of concern in this review. In view of its participation in the most extensive physiological processes and the greatest number of current studies, we have focused on summarizing the metabolism of copper and its interactions with the components of ferroptosis in cancer.^[^ ^24b^ ^]^ Cuproptosis, a new supplementary form of “metalloptosis”, has emerged as an effective strategy for co‐targeting with ferroptosis. The combination of the two might achieve a more ideal effect for cancer therapy (Table 2).^[^ ^7b^ ^]^ Other trace metals, such as zinc, manganese, cobalt, and molybdenum, have been documented to trigger ferroptosis in tumor cells and, similar to copper, are extensively investigated for application in the field of nanomedicine (Table 1). Notwithstanding the common characteristic of these metal ions, which is their ability to trigger Fenton‐like reactions and thereby generate a considerable amount of ROS, it has been ascertained that they possess distinct mechanisms in the regulation of ferroptosis.^[^ ^4^, ^189^ ^]^ However, in contrast to the already familiar forms of ferroptosis regulation, our current knowledge regarding the role of trace metal elements therein remains extremely scarce. Furthermore, other metal elements like calcium might also exert a critical regulatory function in ferroptosis,^[^ ^209^ ^]^ as it is not a trace element, thus it lies beyond the purview of this review.

Despite these substantial accomplishments, numerous issues remain to be addressed in the future with respect to the interaction between trace metal elements and ferroptosis. The following points might deserve consideration: 1) Given that many relevant studies have used metal ionophores or chelators to significantly alter metal concentrations, it is unclear whether the metal content within physiological range would affect ferroptosis; 2) Whether the regulation of ferroptosis by metal elements is indirect and whether it is not the principal one among numerous regulatory factors remains unclear; 3) Whether and in what manner ferroptosis will conversely exert an influence on metal metabolism remains unknown; 4) At present, most research is sporadic and there is a lack of understanding regarding the dependence of different cancers on different metals, which may determine the responsiveness of different cancers to these treatment. Solving this problem is conducive to deepening the comprehension of “metalloptosis”; 5) Although promising achievements have been attained in preclinical studies of metal‐based nanomaterials, the current clinical translation is highly disappointing. A more stable structure, better storability, higher biosafety (that is, minimizing the impact of introducing additional metals on normal human cells), and more durable efficacy are the directions that urgently require addressing.

Collectively, the ongoing research holds the promise of uncovering additional facets of the interaction between metal elements and ferroptosis in cancer, thus facilitating the emergence of new intervention targets. The multidisciplinary cooperation among metal biology, oncology, and materials science will be conducive to the propulsion and development of novel therapeutic approaches for cancer.

## Conflict of Interest

The authors declare no conflict of interest.

## Author Contributions

Xiaoyan Wang: Writing – original draft, review and editing; Preparation of figures; Supervision. Yuanyuan Xue: Writing – review and editing. Lei Chang: Writing – review and editing. Xuena Zhu: Writing – review and editing. Wenjun Liu: Conceptualization; Writing – review and editing; Supervision; Funding acquisition. Tingbo Liang: Conceptualization; Writing – review and editing; Funding acquisition.
